# Light‐Promoted Electrostatic Adsorption of High‐Density Lewis Base Monolayers as Passivating Electron‐Selective Contacts

**DOI:** 10.1002/advs.202003245

**Published:** 2021-01-04

**Authors:** Xi Yang, Zhiqin Ying, Zhenhai Yang, Jia‐Ru Xu, Wei Wang, Jiajia Wang, Zenggui Wang, Lingze Yao, Baojie Yan, Jichun Ye

**Affiliations:** ^1^ Ningbo Institute of Materials Technology and Engineering Chinese Academy of Sciences (CAS) Ningbo 315201 P. R. China; ^2^ Joint Key Laboratory of the Ministry of Education Institute of Applied Physics and Materials Engineering University of Macau Macao SAR 999078 P. R. China; ^3^ 3M China Limited Corporate Research Lab Shanghai 200233 P. R. China

**Keywords:** carrier‐selective contact, electrostatic adsorption, Lewis bases, passivating contact, silicon solar cells

## Abstract

Achieving efficient passivating carrier‐selective contacts (PCSCs) plays a critical role in high‐performance photovoltaic devices. However, it is still challenging to achieve both an efficient carrier selectivity and high‐level passivation in a sole interlayer due to the thickness dependence of contact resistivity and passivation quality. Herein, a light‐promoted adsorption method is demonstrated to establish high‐density Lewis base polyethylenimine (PEI) monolayers as promising PCSCs. The promoted adsorption is attributed to the enhanced electrostatic interaction between PEI and semiconductor induced by the photo‐generated carriers. The derived angstrom‐scale PEI monolayer is demonstrated to simultaneously provide a low‐resistance electrical contact for electrons, a high‐level field‐effect passivation to semiconductor surface and an enhanced interfacial dipole formation at contact interface. By implementing this light‐promoted adsorbed PEI as a single‐layered PCSC for *n‐*type silicon solar cell, an efficiency of 19.5% with an open‐circuit voltage of 0.641 V and a high fill factor of 80.7% is achieved, which is one of the best results for devices with solution‐processed electron‐selective contacts. This work not only demonstrates a generic method to develop efficient PCSCs for solar cells but also provides a convenient strategy for the deposition of highly uniform, dense, and ultra‐thin coatings for diverse applications.

## Graphical abstract

A light‐promoted adsorption method is introduced to establish high‐density Lewis‐base polyethylenimine monolayers, which simultaneously provide a low‐resistance contact for electrons, a high‐quality field‐effect passivation to semiconductor and an enhanced dipole formation at semiconductor/cathode interface, as promising passivating carrier‐selective contacts for solar cells. This work also provides a convenient strategy to deposit highly uniform, dense, and ultra‐thin coatings for diverse applications.

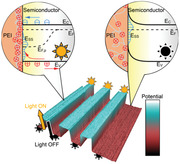

## 1 Introduction

Non‐radiative recombination loss at a semiconductor surface as a result of the electronic contact is one of the main limiting factors to push the performance of photovoltaic (PV) devices closer to the Shockley–Queisser radiative limit.^[^ ^1^, ^2^, ^3^ ^]^ When contacting the semiconductor with charge extracting contacts, the additional parasitic recombination is generally induced by the defects or surface trap states in the forbidden gap because of the presence of material imperfections, such as unsaturated surface dangling bonds due to the broken periodicity typically for covalently bonded inorganic semiconductor, under‐coordination ions typically for weakly bonded (i.e., ionic bonds, hydrogen bonds, and van der Waals interactions) organic semiconductor, or attached impurity. This will reduce the quasi‐Fermi level splitting as well as the effective charge densities, and ultimately the attainable open‐circuit voltage (*V*_OC_) and short‐circuit current (*J*_SC_).^[^ ^4^, ^5^, ^6^ ^]^ To eliminate this contact‐induced recombination issue, the passivating carrier‐selective contacts (PCSCs), which effectively suppress charge recombination through surface defect states and simultaneously function as contacts to selectively conduct one type of carriers while block the other type, are recently proposed and widely investigated.^[^ ^7^, ^8^, ^9^ ^]^ In general, ideal PCSCs should fulfill several requirements including 1) low‐contact resistivity to the collected carrier, 2) effective passivation of defects typically present at the semiconductor surface, 3) well‐matched energy‐band alignment to facilitate the carrier ejection, 4) broadband transparent to avoid the parasitic absorption loss, and 5) simple deposition method at low thermal budget.

Historically, many PV technologies, such as silicon, III−V, copper indium gallium selenide, perovskite (PVK), and organic solar cells, had been successfully optimized for higher performance by focusing on strategies towards the implementation of efficient electron‐selective materials, including alkali/alkaline earth metal salts, metal oxides, and nitrides, organic polymers, low work function (WF) metals, and their combinations.^[^ ^10^, ^11^, ^12^, ^13^, ^14^, ^15^, ^16^, ^17^, ^18^, ^19^, ^20^, ^21^, ^22^, ^23^ ^]^ However, the main challenge in using these electron‐selective contacts is their poor surface passivation of semiconductor surfaces, which in turn decreases their carrier selectivity and results in a relatively low efficiency when applied at the device level. For this reason, most of them require either an additional passivation layer, such as intrinsic hydrogenated amorphous silicon (a‐Si:H), silicon dioxide, and aluminum oxide for silicon solar cells, polystyrene, poly(methyl methacrylate), and lithium fluoride for perovskite solar cells, or an extra processing step, such as annealing and hydrogenation treatments, to realize the passivating contacts, both of which may bring in excessive resistance or parasitic absorption losses and limit their industrial applications. Therefore, it is still challenging to achieve both an efficient carrier‐selectivity and a high‐level passivation in a sole interlayer without any complicated procedure.

Recently, several studies have demonstrated that the electronic trap states at the semiconductor surface can be significantly passivated by using the organic materials with Lewis base or acid functional groups, such as thiophene, pyridine, carbonyl, amine, and sulfonate.^[^ ^24^, ^25^, ^26^, ^27^, ^28^, ^29^ ^]^ For covalent semiconductors (e.g., Si, Ge), the Lewis base (or acid) groups withdraw electrons from (or donate electrons to) the semiconductor, remaining positive (or negative) fixed charges at the surface and establishing an electric field to provide a field‐effect passivation by repelling the holes (or electrons) from the surface region.^[^ ^29^ ^]^ For ionic semiconductors (e.g., PVK, GaAs), these Lewis base (or acid) groups, performing as the electron donors (or acceptors), can directly bind to the positively (or negatively) charged under‐coordinated ions and thus chemically passivate the ions induced traps states through the formation of Lewis adducts.^[^ ^24^, ^30^ ^]^ Also, these Lewis base and acid materials are generally featured by polar moiety, which can induce interfacial dipoles to achieve appropriate energy level matching at the contact interface and thus minimize the energy barrier for carrier‐selective extraction.^[^ ^31^, ^32^, ^33^ ^]^ Therefore, benefiting from these multifunctional groups, the Lewis base and acid based organic carrier transporting layers are expected to simultaneously possess promising surface passivation and carrier‐selectivity.

In this framework, electrostatically self‐assembled monolayer of polyethylenimine (PEI) can be considered as a valid alternative to conventional passivating electron‐selective layers. The PEI, as a cationic polyelectrolyte with a high content of Lewis base amines, can be physisorbed from solution onto various surfaces and form extremely thin (monolayer‐scale) and stable layers, by low‐temperature and solution‐processed methods such as spin‐, blade‐, dip‐, spray‐, and slot‐die‐ coating.^[^ ^34^, ^35^, ^36^ ^]^ Besides its facile deposition, the main advantage of PEI is the ability to modify the electrical properties of the surface on which it is attached. On one hand, the amines in PEI chains can donate nitrogen lone pairs to the semiconductor, thus providing the field‐effect surface passivation by repelling holes from the surface as well as the chemical passivation by binding to the under‐coordinated cationic surface states.^[^ ^29^ ^]^ On the other hand, the molecular dipole associated with the amines, and the interfacial dipole associated with the charge‐transfer at the contact interface, together can substantially reduce the WF of a wide range of substrates such as metals, metal oxides, conducting polymers, and graphene.^[^ ^32^ ^]^ Although the PEI has been widely applied in silicon, organic, PVK, and tandem solar cells,^[^ ^37^, ^38^, ^39^, ^40^ ^]^ however, to the best of our knowledge, it is rarely used as a passivating electron‐selective contact on its own (without additional passivating layer). This is mainly because of the thickness dependence of the contact resistivity and the passivation quality in the PEI interlayer, both of which increase with film thickness. Typically, the PEI interlayer should be as thin as possible to implement the carriers tunneling or hooping,^[^ ^41^ ^]^ because the electrical insulating nature of thick PEI can attenuate its function as a carrier‐selective layer or make it function just as a resistive blocking layer without any carrier‐selectivity.^[^ ^42^ ^]^ However, when the PEI interlayer is too thin, the semiconductor surface is difficult to be completely covered by the isolated adsorbed coil‐like PEI chains, which makes it more prone to be Fermi‐level pinned by the occurrence of metal‐induced gap states (MIGS) due to the direct contact between semiconductor and metal after metallization.^[^ ^43^ ^]^ In addition, relatively less amount of amines in ultra‐thin PEI is insufficient for the repelling of holes from surface or for the neutralization of ions induced traps states, thus resulting in an ineffective field‐effect or chemical passivation. Moreover, because of the weakly charged semiconductor surface and the inevitably short‐range Columbic repulsion between the PEI chains, the ultra‐thin PEI monolayer adsorbs rather sparsely and inhomogeneously.^[^ ^44^ ^]^ The derived less density of amines as well as higher density of pinholes in the monolayer (especially when the monolayer thickness is less than the pinhole diameter) will limit the strength of both molecular and interfacial dipole, and result in a degenerative WF modulation and thus a poor electron‐selectivity.^[^ ^45^, ^46^ ^]^ On this basis, a new strategy to realize low‐temperature and solution‐processed ultra‐thin PEI monolayers with high‐density Lewis base amines and uniform film coverages is urgently needed for achieving efficient passivating electron‐selective contacts with both low contact resistivity and high level of passivation.

Herein, we show that the adsorption of PEI on semiconductor surface can be significantly promoted purely by simple optical means. This is achieved by exploiting the surface potential shift of the semiconductor caused by the injection of photo‐generated nonequilibrium carriers that is decisive of the extent of electrostatic interaction between negative *n‐*type semiconductor surface and positive PEI segments. As compared to the conventional PEI layers deposited without illumination, the light‐promoted adsorbed PEI layer exhibited a decreased film thickness but an increased amount of amines and an improved surface coverage. These high‐density Lewis base amines in the angstrom‐scale PEI monolayer are demonstrated to simultaneously provide a low‐resistance electrical contact for electrons (i.e., contact resistivity ≈ 26.7 mΩ·cm^2^), a moderate field‐effect passivation to *n*‐type silicon (*n*‐Si) surface (i.e., recombination current density ≈ 387 fA cm^−2^) and an enhanced dipole (i.e., WF ≈ 3.34 eV) formation at the interface between *n*‐Si and Al. The derived high‐performance PCSC is then applied as a full‐area rear contact in the proof‐of‐concept *n*‐Si solar cell, for the first time using the PEI on its own, achieving a power conversion efficiency (PCE) of 19.5% with a *V*_OC_ of 0.641 V, a *J*_SC_ of 37.6 mA cm^−2^ and a fill factor (*FF*) of 80.7%, which is one of the best results for devices with solution‐processed electron‐selective contacts. These results open up the possibility of using light‐promoted electrostatically adsorbed angstrom‐scale organic monolayers as efficient PCSCs in PV devices not only for silicon but also for PVK and organic solar cells, due to their solution‐processed and annealing‐free fabrication, as well as outstanding optoelectronic properties. Furthermore, the study also provides a convenient and effective strategy for the deposition of highly uniform, smooth, dense, and ultra‐thin coatings for various applications.

## 2 Results and Discussion

### 2.1 Mechanism for the Light‐Promoted Electrostatic Adsorption

**Figure 1:**
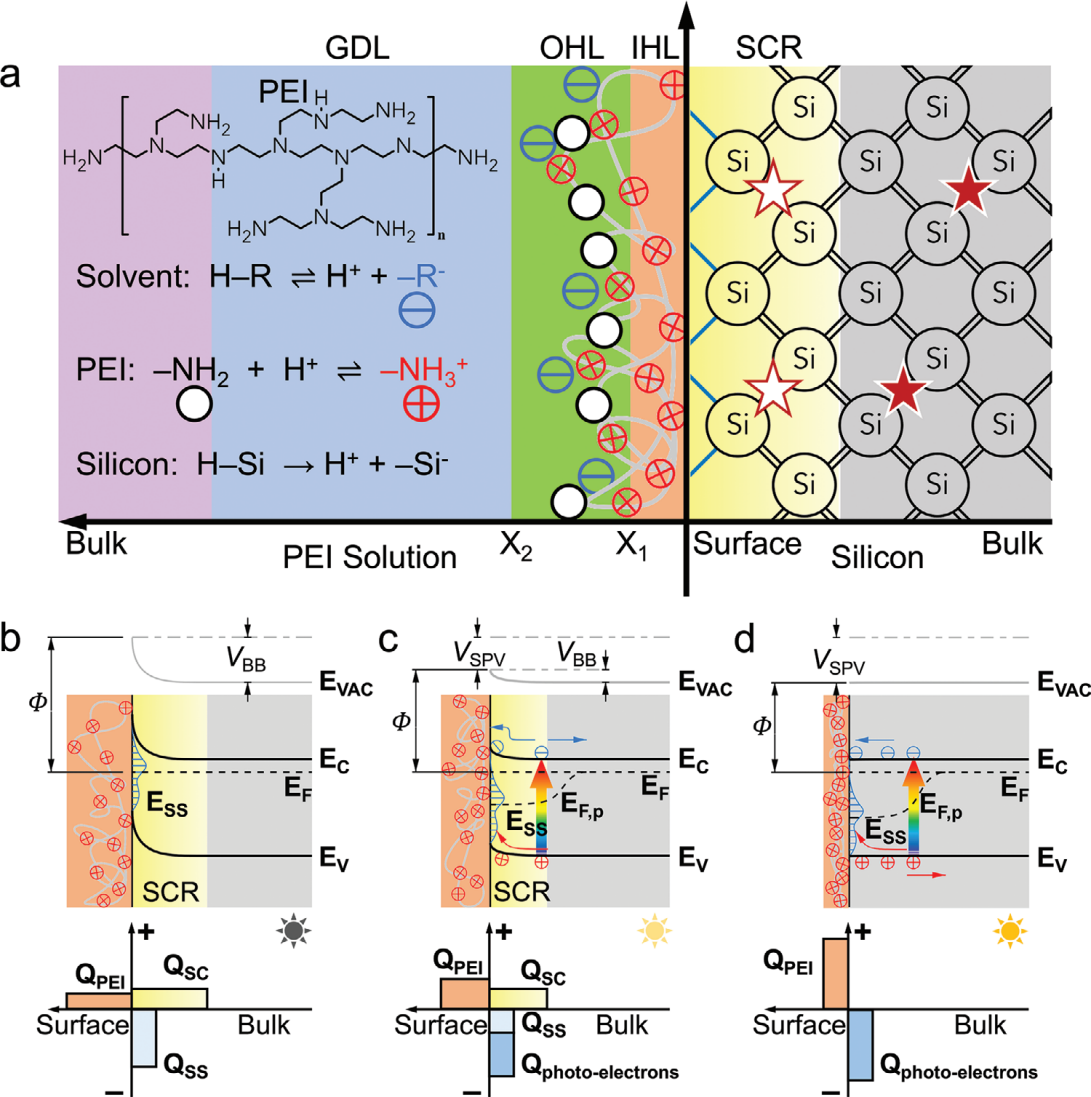
The basic concepts of the PEI adsorption on *n*‐Si surfaces with and without illumination. a) The double‐layer structure model of the *n*‐Si in contact with the PEI solution under equilibrium conditions. Six different lateral regions exist in the *n*‐Si–PEI solution system. They are, from left to right, the bulk of PEI solution (purple); the Gouy diffuse layer (GDL, blue); the outer Helmholtz layer (OHL, green); the inner Helmholtz layer (IHL, orange); the space‐charge region (SCR, yellow); the semiconductor bulk (gray). The stars represent dopants, which are ionized in the SCR. The gray line and black, red, and blue circles in Helmholtz layers represent the adsorbed PEI chains, neutral amines, protonated amines, and counter anions, respectively. Band diagram of the *n*‐Si in equilibrium with the PEI solution b) in dark condition, c) under illumination and d) in the case of the photosaturation. The energy level of vacuum, valence band, conduction band, surface states as well as the Fermi level and surface WF are denoted by *E*_VAC_, *E*_V_, *E*_C_, *E*_SS_, *E*_F_ and *Φ*, respectively. The *Q*_SS_, *Q*_SC_, *Q*_PEI_ and *Q*_photo‐electrons_ denote the charges of surface trap states, SCR, PEI, and photogenerated nonequilibrium electrons, respectively. The *V*_BB_ is the potential barrier for the movement of electrons from bulk to surface due to the surface band bending. The *V*_SPV_ is the surface photovoltaic induced by the illumination.

To understand the electrostatic interactions between the Lewis base solution and the semiconductor surface, we first focus on the adsorption process of PEI on *n*‐Si. For this, the Derjaguin–Landau–Verwey–Overbeek theory is used for modeling all the interactions in the system so as to get more insight into the entire adsorption processes.^[^ ^47^ ^]^ As an adsorbate, the PEI is one type of Lewis bases providing functional amines (–NH*_x_*) as electron‐donating end groups, which can be partially protonated by accepting protons dissociated from the polar solvents such as water and ethanol (–NH*_x_* + H^+^ = –NH*_x_* ^+^), leaving positive‐charged segments on polymer chains and releasing counterions in solution.^[^ ^37^ ^]^ As an adsorbing substrate, the hydrogen‐terminated *n*‐Si (HF treated) in solution exhibits a net negative surface charge (–SiH = –Si^‐^ + H^+^) due to the existence of electronically active states at surface.^[^ ^48^ ^]^ When contacting with the PEI solution, the *n*‐Si surface provokes an interfacial charge redistribution that is different from the charge distribution in the liquid phase. To neutralize the *n*‐Si surface charge, the positive charges of PEI segments in solution combined with the uncompensated negative charges on *n*‐Si electrostatically induce the formation of an electrical double layer (EDL) which is made up of several layers (**Figure** **1**a). The first layer, named as the inner Helmholtz layer (IHL), contains positive‐charged amines and oriented solvate molecules. The center of electrical charge occurs at a distance *X*_1_ from the Si surface. The next layer is called the outer Helmholtz layer (OHL) and consists of solvated ions, neutral amines and counter anions which can approach the *n*‐Si surface to a distance *X*_2_. Next to the OHL is the Gouy diffuse layer (GDL) which extends from the OHL into the bulk of the solution. It is filled with numerous cations and few anions. Therefore, the total driving force in the adsorption process is determined by the balance between electrostatic attraction (*W*_att_) of charged PEI segments to the *n*‐Si and the repulsion (*W*_rep_) in PEI segment‐segment (see details in Note S1, Supporting Information). For weakly charged surface, the amount of adsorbed PEI is limited by the poor *W*_att_, and the chains adopt a thick and swollen‐coil conformation due to the dominant *W*_rep_, resulting a looser molecular packing. While for strongly charged surface, where the *W*_rep_ is screened by the dominant *W*_att_, a higher amount of adsorbed PEI amines is needed to compensate the surface charges of *n*‐Si, and the chains adsorb in a thinner and flat‐on conformation, resulting a denser molecular packing. As a consequence, it is anticipated that alterations to the surface potential of *n*‐Si can significantly influence the amount, the thickness, the conformation and thus the density of the adsorbed PEI.

The strategy of light induced charge transfer, as a feasible method to manipulate the surface potential of semiconductor, has been widely studied for applications in various optoelectronic devices such as solar cells, photoelectrochemical cells, photodetectors, and generator.^[^ ^49^, ^50^, ^51^, ^52^ ^]^ Here, we further analyze the influence of illumination on the adsorption of PEI on *n*‐Si. Under dark condition, the Fermi level (*E*_F_) of the bulk *n*‐Si aligns with the energy level of the surface trap states (*E*_SS_) to achieve electronic equilibrium, depicted in Figure 1b as upward band bending (BB) of the vacuum level (*E*_VAC_), forming a depleted space‐charge region (SCR) full of immobile ionized donors. When the *n*‐Si surface is contacted with the PEI solution, the applied solution potential is accommodated by a corresponding change in the potential drop across the EDL, whereas the Fermi level on *n*‐Si surface stays virtually fixed and the degree of BB remains constant due to the Fermi level pinning.^[^ ^53^ ^]^ As a result, because of the long‐range nature of the electrostatic interaction, these positive charges in SCR (*Q*_SC_) compensate the negative charges at surface trap states (*Q*_SS_) and thus hinder the PEI adsorption (*Q*_PEI_ = *Q*_SS_ – *Q*_SC_). Under illumination, the photogenerated carriers in *n*‐Si will redistribute under the influence of the space charge field. This results in a potential gradient for photogenerated electrons to move towards the bulk, and for photogenerated holes to move towards the surface (Figure 1c). Upon increasing the illumination intensity, the Fermi level pinning will be gradually released (known as photoinduced passivation) due to the screening of *Q*_SS_ by photogenerated nonequilibrium holes (*Q*_photo‐holes_), resulting a decreased level of surface BB (see details in Note S2, Supporting Information). Under sufficient intensity of illumination (so called photosaturation), flat band condition (Figure 1d) can be achieved when the *Q*_photo‐holes_ matches the *Q*_SS_ (*Q*_photo‐holes_ = *Q*_SS_) due to the completely screened SCR (*Q*_SC_ = 0). As a result, the photogenerated nonequilibrium electrons (*Q*_photo‐electrons_) are more prone to be drawn towards the surface under the attraction of positive‐charged PEI, rather than to diffuse back to the bulk, which can also contribute to the net surface charges. As a response, more protonated amines in the PEI solution would migrate into the EDL to compensate the *Q*_photo‐electrons_ (*Q*_PEI_ = *Q*_photo‐electrons_), thus boosting the PEI adsorption.

These theoretical analyses reveal that the interaction between the *n*‐Si surface and the PEI segment is a critical factor for the PEI adsorption, and the adsorption behavior can be greatly influenced by the amount of negative charges on *n*‐Si surface. Under dark conditions, the amount of negative charges on *n*‐Si surface is limited by the space charge field and the upward BB. Whereas the illumination screens the space charge field and decreases the BB by depinning the Fermi level, and concomitantly generates a large amount of nonequilibrium electrons, which can successively participate in adsorbing PEI molecules onto the surface. Moreover, the increase in charge density at the interface also decreases the surface tension, which in turn reduces the critical free energy of the PEI nucleation and thus improves the surface coverage of the adsorbed PEI.^[^ ^54^ ^]^ Therefore, we can expect that applying the illumination onto the semiconductor can be an efficient strategy to promote the adsorption of PEI and thus to increase the density of the adsorbed PEI.

### 2.2 Light‐Promoted Electrostatic Adsorption for High‐Density PEI Monolayers

**Figure 2:**
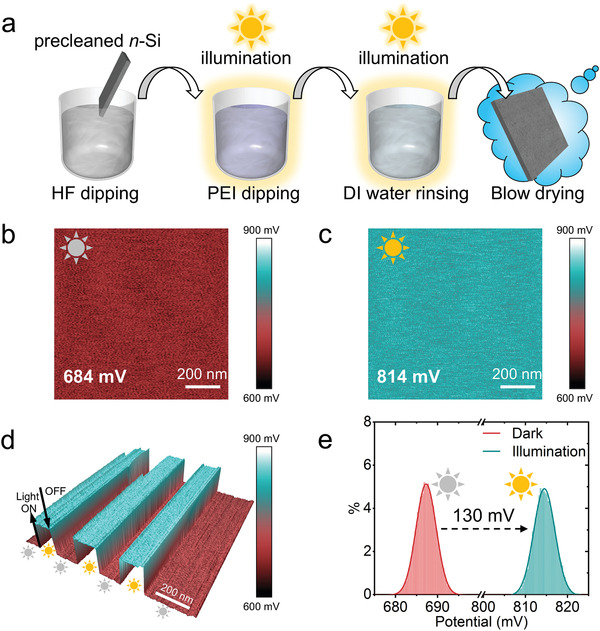
The light induced surface photovoltage of the *n*‐Si. a) Schematic illustration of the procedure for light‐promoted electrostatic adsorption of PEI on *n*‐Si surface. 2D Scanning Kelvin probe microscopy (SKPM) image of *n*‐Si b) in dark and c) under illumination. d) 3D SKPM image showing instantaneous photoresponse when three light ON/OFF cycles were triggered, which results in distinct bright and dark bands corresponding to the cycles of light ON and OFF. e) Potential histograms corresponding to the bright and dark scans. The peak‐to‐peak distance gives the average potential shift.

In order to test our proposed mechanism for light‐promoted electrostatic adsorption, we applied the illumination to the *n*‐Si substrate during the PEI adsorption. The schematic illustration of the procedure for light‐promoted electrostatic adsorption is presented in **Figure** **2**a. Following a quick immersion in diluted HF solution for native oxide removal, the precleaned *n*‐Si substrate was dipped into a PEI solution for 1 min under a super‐bandgap illumination. Then, an intermediate water washing was performed for 1 min with gentle shaking in deionized (DI) water to rearrange the adsorbed PEI monomers and remove the loosely‐attached PEI monomers.^[^ ^55^ ^]^ Subsequently, the remnant solvent was gently dried by nitrogen flow to yield an ultra‐thin PEI monolayer. (Details can be found in the Experimental Section.) It is of note that the dip‐coating method, which does not need expensive precision instruments, is fast‐processing and compatible with mass production. Moreover, compared with the conventional spin‐coating method, this simple dip‐coating is more suitable for large‐area application and conformal coating of textured or rough substrates.^[^ ^56^ ^]^

To corroborate the photoinduced decrease in the space charge field and flattening of the BB on the *n*‐Si surface, we performed scanning Kelvin probe microscopy (SKPM) measurements to gauge the surface photovoltage (*V*_SPV_) of the *n*‐Si induced by illumination, which can be defined as the difference between the values of surface potential in the dark (*φ*_dark_) and under illumination (*φ*_ill_). It should be noted that the value of the measured potential is not the absolute value but the potential of the surface relative to the probe tip, that is, the contact potential difference (*V*_CPD_). Illuminating the sample under scan with a white light of 5000 lux (Figure 2c) shows a distinct *V*_CPD_ shift as opposed to that under dark scan (Figure 2b). To check the spontaneity of *V*_CPD_ shift under illumination, three light ON/OFF cycles were triggered in the course of the SKPM scan, resulting in a *V*_CPD_ map with distinct bright and dark bands corresponding to the cycles of light ON and OFF, respectively (Figure 2d). A large *V*_CPD_ shift from 684 mV for dark scans to 814 mV for bright scans can be clearly determined from the *V*_CPD_ histograms (Figure 2e), indicating the positive *V*_SPV_ as well as the down‐ward BB induced by the illumination (Figure S1 and Note S3, Supporting Information). Since the *V*_CPD_ variation is photoinduced, it is intriguing to quantify the effect of the illuminance on the *φ*_surface_ shift. We vary the illuminance from ≈0 to 5000 lux during the SKPM scans and achieve a photosaturation condition at the illuminance above 5000 lux (Figure S2, Supporting Information). Henceforth, we chose two sets of illuminance with ≈0 lux (only indoor light without additional illumination) as a control (referred to as dark‐PEI) and 5000 lux for comparison (referred to as ill‐PEI), to investigate the effects of illumination on the PEI adsorption.

**Figure 3:**
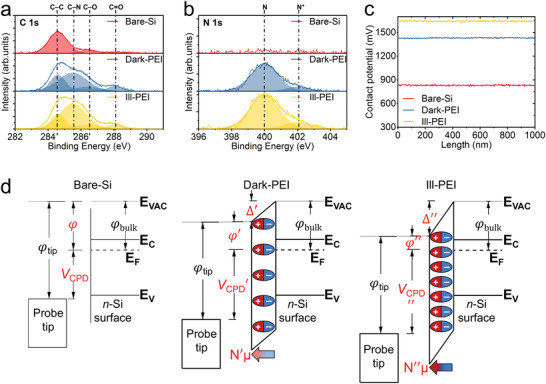
Density enhancement in the PEI monolayer adsorbed under illumination. High‐resolution X‐ray photoelectron spectroscopy spectra of a) C 1s and b) N 1s core level of bare Si, dark‐PEI/Si and ill‐PEI/Si. c) Contact potential profiles (*V*_CPD_) extracted from the scanning kelvin probe microscopy images (Figure S6, Supporting Information) of bare Si, dark‐PEI/Si and ill‐PEI/Si. d) Proposed *V*_CPD_ variation scheme originating from dipole formation induced by adsorbed PEI on *n*‐Si. The introduction of the PEI layer results in a down‐shift of *E*_VAC_ by the formation of the interfacial dipole pointing outward the Si surface and increases the *V*_CPD_, which is proportional to the magnitude (∆) of the generated dipole. N and µ is the density and moment of the dipole in PEI, respectively.

To further verify whether the photo‐generated electron–hole pairs enhance the PEI adsorption, we evaluated the surface chemistries of the dark‐ and ill‐ PEI adsorbed on *n*‐Si substrates, using X‐ray photoelectron spectroscopy (XPS) measurements. For comparison, a fresh HF‐treated Si substrate without PEI adsorption (bare‐Si) was used as a reference sample. The survey XPS spectra of these three samples are illustrated in Figure S3, Supporting Information. The atomic concentrations of various principal elements (C, N, O, and Si) are listed in Table S1, Supporting Information. The occurrences of N element confirm the presence of PEI on *n*‐Si surfaces for both dark‐PEI and ill‐PEI samples. Concomitantly, the composition of Si element decreases, suggesting that signal from the *n*‐Si substrate is obscured upon the PEI adsorption. The atomic concentration of Si element decreases from 53.1 for bare‐Si to 38.5 for dark‐PEI and 42.2 for ill‐PEI, while the atomic concentrations of N element increases to 5.1 and 6.7, respectively. As we known, a higher concentration of N element indicates a larger amount of PEI monomer and a higher concentration of Si element implies a thinner film thickness of adsorbed layer.^[^ ^57^ ^]^ Hence, we conclude that the illumination not only increases the amount of the adsorbed PEI monomers but also decreases the thickness of the adsorbed PEI layer, thus confirming the occurrence of the light‐promoted adsorption of high‐density PEI monolayers with a high level of amines coverage. For clarity, the fine XPS spectrums of C 1s and N 1s have been fitted by multiple Gaussians (see details in Note S4, Supporting Information). The peak area ratio of C−N (285.6 eV)/C−C (284.6 eV) increases from 2.2 for dark‐PEI to 3.1 for ill‐PEI (**Figure** **3**a), also attesting that more PEI monomers are adsorbed under the illumination. The N 1s peak intensity of the ill‐PEI at high BE (with a [N^+^]/[N] ratio of 15.4) is ≈141 % greater than that of the dark‐PEI (with a [N^+^]/[N] ratio of 10.9), which means more amines are protonated to neutralize the photo‐generated electrons on illuminated *n*‐Si surface during adsorption (Figure 3b). Moreover, the influence of the illumination on the thickness of the adsorbed PEI layer on *n*‐Si surface is further estimated from the attenuation of the Si 2p intensities (Figure S4 and Note S5, Supporting Information). The obtained values for the thickness of adsorbed PEI with and without illumination are 9.1 and 12.7 Å, respectively, consistent with the values obtained from the ellipsometry measurements.

Topographical atomic force microscopy (AFM) measurements were then carried out to evaluate the influence of illumination on the surface morphology and the chain conformation of the adsorbed PEI monolayers. Because of the self‐adjusting capability originated from the soft nature of PEI chains,^[^ ^58^ ^]^ the root mean square (RMS) roughness decreases from 3.34 Å for the initial bare‐Si to 2.22 Å for dark‐PEI monolayer. The AFM height images and the profilometric height line scans (Figure S5, Supporting Information) reveal the presence of small dispersed aggregates with 1.2 nm in height and diameter ranging between 300 and 500 nm, suggesting a swollen‐coil conformation as well as a poor coverage of the adsorbed dark‐PEI. However, in the case of the ill‐PEI monolayer, a remarkably smooth surface with a reduced RMS roughness of 1.96 Å is observed, attesting to a homogeneous coverage of the adsorbed ill‐PEI. Such a low film thickness as well as the uniform appearance of ill‐PEI appear to reflect a strongly charged *n*‐Si surface induced by the photo‐generated carriers, which increases the electrostatic attraction to PEI and forces the PEI chain conformation to align flatly with the surface.^[^ ^44^ ^]^

Given that the WF of semiconductor surface can be modified by the adsorption of positive‐charged PEI monolayer, the SKPM measurements were performed to assess the effect of illumination in terms of *V*_CPD_ (Figure S6, Supporting Information). The change in *V*_CPD_ values is commonly used to estimate the variation of the surface dipole, WF and surface wetting properties, thus here can be related to the density of amines within the adsorbed PEI films. As shown in Figure 3c, the *V*_CPD_ is found to increase form 830 mV for bare‐Si to 1420 mV after the adsorption of dark‐PEI. This positive shift of *V*_CPD_ for the PEI modified sample indicates a down‐shift of *E*_VAC_ compared to that for the bare‐Si, consistent with the SKPM measurements by others on similar amines grown on ITO.^[^ ^59^ ^]^ As expected, the illuminated adsorption leads to a further increase in *V*_CPD_ to 1640 mV for ill‐PEI. Such a behavior can be explained by the charge rearrangement between PEI and *n*‐Si substrate. The amines in PEI behaving like surface donors donate electrons to *n*‐Si and leave the PEI partially positive‐charged while the outermost atoms of *n*‐Si negative‐charged, creating an interfacial dipole directed away from *n*‐Si and resulting a down‐shift *E*_VAC_ as well as an increased *V*_CPD_ (Figure 3d). Therefore, the largest *V*_CPD_ is a good indicator of the highest density of adsorbed amines in ill‐PEI sample (see details in Note S6, Supporting Information). This is further corroborated by the contact angle (CA) measurement (Figure S7, Supporting Information) which shows that the ill‐PEI with the highest density of hydrophilic amines has the lowest value of CA (Note S7, Supporting Information), also in good agreement with the results of XPS measurements. All of the experimental evidences presented thus far collectively indicate that the adsorption of PEI on *n*‐Si processed with illumination is significantly enhanced in terms of a reduced film thickness, an increased amines density as well as an improved film coverage.

### 2.3 Passivating Electron‐Selective Characteristics of Light‐Promoted Adsorbed PEI Monolayers

**Figure 4:**
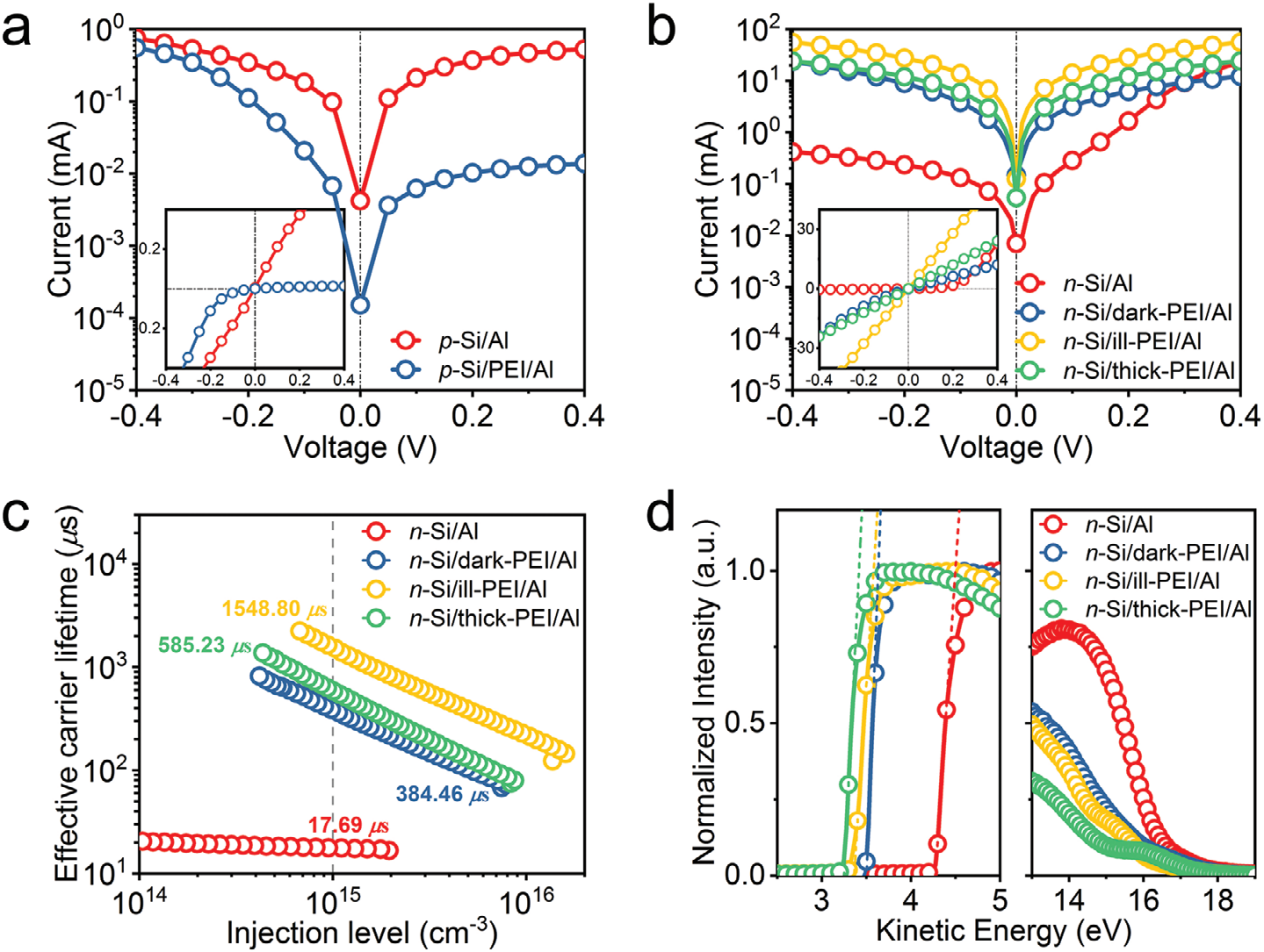
Electrical properties of the *n*‐Si/ill‐PEI heterocontacts. The semilogarithmic dark *J*–*V* curves for a) *p‐*Si/Al and b) *n*‐Si/Al contacts with and without PEI interlayers. The insets show the same data on a linear scale. c) Value of the effective carrier lifetime versus injection level measured for *n*‐Si/Al contacts with different PEI interlayers. d) The normalized UPS spectra of *n*‐Si/Al contact interfaces with different PEI interlayers. The secondary electron cutoff region is displayed with the kinetic energy on the abscissa to directly show the WF of the sample.

Having confirmed that the illumination is, in fact, able to promote the adsorption of PEI on *n*‐Si, we move on to probe the effects of this developed ill‐PEI on the optoelectronic properties of *n*‐Si based solar cells. Before the application of the ill‐PEI to actual devices, a series of electrical heterocontacts with different structures were fabricated to exploit the beneficial effects of the high‐density amines on the electron selectivity. The test structure is schematically depicted in Figure S8, Supporting Information. The contact based on conventional spin‐coated PEI layer (hereafter referred to as thick‐PEI) was studied for comparison. The resulting dark log‐plot current density (*J*)–voltage (*V*) curves at room temperature are presented in **Figure** **4**a,b. For the *p‐*Si substrate (Figure 4a), where holes carry most of the current, the *J*–*V* characteristic exhibits ohmic behavior with a small rectification ratio (*K* = |*J*(‐0.4 V)/*J*(0.4 V)|) of 1.4 for the *p‐*Si/Al contact because of a small barrier height between Al and *p‐*Si. However, by inserting the PEI monolayer between Al and *p‐*Si, the *J*–*V* curve exhibits a non‐ohmic behavior with an increased *K* of 40.2, demonstrating the hole‐blocking property of the Si/PEI/Al heterocontact.^[^ ^16^ ^]^ For the *n*‐Si substrate (Figure 4b), where electrons carry most of the current, the *J*–*V* characteristic of the *n*‐Si/Al contact exhibits rectifying behavior with a large *K* of 54.3, suggesting the presence of a large Schottky barrier height (*Φ*_B_) between *n*‐Si and Al caused by MIGS. When the dark‐PEI is inserted between the *n*‐Si and Al, the contact exhibits a much reduced *K* of 1.96, and the *J*–*V* relation is nearly Ohmic (linear, see inset to Figure 4b) rather than exponential under forward bias, attesting the reduction in the effective *Φ*_B_ brought by the amines in dark‐PEI.^[^ ^60^ ^]^ In contrast, the *J*–*V* curves for the *n*‐Si/thick‐PEI/Al and *n*‐Si/ill‐PEI/Al contacts exhibit perfectly linear, ohmic contact characteristics (*K* ≈ 1) within the probed current density range, implying that both two samples contain large amounts of amines and can completely eliminate the *Φ*_B_. As the total contact resistivity (*ρ*_c_) of this metal‐insulator‐semiconductor (MIS) contact can be divided into the contribution from the resistance associated with the *Φ*_B_ between the metal and the semiconductor (*R*_B_) in parallel with the tunneling resistance (*R*_T_) of insulator,^[^ ^61^ ^]^ we attributed the larger *R*_T_ within the thick thick‐PEI interlayer as the main cause for the smaller current under both positive and forward voltage biases. The *ρ*_c_ were further extracted by the Cox and Strack method.^[^ ^62^ ^]^ By fitting the trend of resistance versus front circular contact diameter, we extracted a low *ρ*_c_ of 26.7 mΩ·cm^2^ for *n*‐Si/ill‐PEI/Al contact, which is almost ten times lower than that of *n*‐Si/thick‐PEI/Al contact (250 mΩ·cm^2^) and is much lower than that of the electron‐selective MgF*_x_*, Mg, TaO*_x_*, or TaN*_x_* contacts on an *n*‐Si substrate (is comparable to that of the electron‐selective TiO*_x_* and MgO*_x_* contacts, see Table S2, Supporting Information). These results demonstrate that the *n*‐Si/ill‐PEI/Al stack has excellent properties to act as an efficient electron‐selective and hole‐blocking contact for high‐efficiency solar cells.

A high‐performance PCSC requires not only a low contact resistivity at the contact interface but also a good surface passivation that enables a low carrier recombination velocity at the semiconductor surface. To gain a better understanding into the passivation effect of the high‐density amines in ill‐PEI, the recombination at the interface between *n*‐Si and adsorbed PEI was evaluated via effective carrier lifetime measurements on symmetrically film‐coated sample (see a schematic of the test structure in Figure S9, Supporting Information). As can be seen in Figure 4c, the effective carrier lifetime (*τ*_eff_) and the recombination current density (*J*_0_) a function of excess carrier density Δ*n* were measured using the quasi‐steady state photoconductance (QSSPC) technique.^[^ ^63^ ^]^ For the direct contact of *n*‐Si with Al, due to the Fermi level pinning effect, an internal electric field induced by the MIGS promote the movement of minority‐carriers (holes) toward the surface (Figure S10a, Supporting Information). In this case, the holes easily recombine at the surface under illumination, resulting a poor *τ*_eff_ of 17.69 *µ*s and a high *J*_0_ of 1900 fA cm^−2^. However, for the contact of *n*‐Si/dark‐PEI/Al, the dark‐PEI interlayer enabled the *τ*_eff_ of up to 384.46 *µ*s (with a *J*_0_ of 1420 fA cm^−2^), indicating that the positive amines in dark‐PEI create a reversed electric field which can neutralize the MIGS induced electric field and hinder the movement of holes toward the surface (Figure S10b, Supporting Information). As expected, a longest *τ*_eff_ of 1548.80 *µ*s (with a *J*_0_ of 387 fA cm^−2^) is obtained for the contact of *n*‐Si/ill‐PEI/Al, which is even 2.6× higher than those obtained for thick‐PEI (with a *τ*_eff_ of 585.23 *µ*s and a *J*_0_ of 1000 fA/cm^2^), attesting to a furtherly enhanced reversed electric field induced by the high‐density amines in ill‐PEI (Figure S10c, Supporting Information). In this case, the ill‐PEI with a large amount of positive amines acts as a minority‐carriers mirror repelling most of holes form the *n*‐Si surface and turns the space‐charge region into accumulation (downward BB), thus significantly suppressing the surface recombination as the arrival rate of minority‐carriers at recombination centers is the rate determining step in surface recombination. It is worth noting that, although additional a‐Si:H passivation layers have been recently reported to further improve the *τ*_eff_ to 2.4 ms for a‐Si:H/PEI contacts, such a multilayered structure inevitably suffers from an increased *ρ*_c_ due to the additional a‐Si:H layer induced bulk resistance (Table S2, Supporting Informantion).^[^ ^40^ ^]^ In addition, the vacuum and relatively high‐temperature plasma‐enhanced chemical vapor deposition (PECVD) process for the fabrication of a‐Si:H also impede efforts to further reduce costs.

Since the recombination process in a semiconductor requires the presence of both recombination centers and carriers, a long *τ*_eff_ can be achieved by reducing the number of defect states (chemical passivation) and/or one type of carriers available at the surface (field‐effect passivation).^[^ ^8^ ^]^ As shown in a control experiment (Figure S11, Supporting Information), when further capping a poly(4‐styrenesulfonic acid) (PSS) layer with negative‐charged sulfonic acid groups, which neutralized the positive amines in PEI but preserved the initial interface between the PEI and *n*‐Si,^[^ ^64^ ^]^ the *τ*_eff_ decreases back to 190.38 *µ*s. Thereby, this passivation reversibility implies that the increased *τ*_eff_ in PEI sample is mainly due to the field‐effect passivation rather than the chemical passivation. The passivation stability was also investigated on the *n*‐Si/ill‐PEI/Al contact. When exposed to air, the sample passivated by ill‐PEI exhibits a *τ*_eff_ stability much better than that of HF‐treated samples (Figure S12, Supporting Information). The *τ*_eff_ of the ill‐PEI passivated sample reduces to ≈40% (from 1548.80 *µ*s to 680.49 *µ*s) after 24 h air exposure, whereas *τ*_eff_ of HF‐treated sample sharply drops to only 1% (from 1460.12 to 15.15 *µ*s) due to the natural oxidation of the Si surface. We attribute this passivation stability of *n*‐Si/ill‐PEI/Al contact to the high reductive capability of PEI,^[^ ^65^ ^]^ which can primitively protect the Si surface from being oxidized during the photo‐irradiation process. These results demonstrate that the high‐density amines in ill‐PEI can provide excellent field‐effect passivation to *n*‐Si, giving thereby the contact its desired carrier selectivity.

In order to gain insight into the passivating electron‐selective properties of the *n*‐Si/ill‐PEI/Al contact, we subsequently investigate the band alignment at the contact interface. For the single contact of metal/PEI (Figure S13a, Supporting Information) and semiconductor/PEI (Figure S13b, Supporting Information), because of the strong electron‐donor nature of nitrogen lone pairs in amines, the electron transfer from PEI to semiconductor or metal renders the vacuum side positive‐charged. The PEI layer change the intrinsic surface potential of the solid by interacting with the “‘free”’ electron wave function of the solid which extends slightly outside the surface, creating a dipole with its negative pole pointing outwards.^[^ ^66^ ^]^ The electron density of PEI normally acts to “‘push back”’ the solid wave function and, therefore, reduces the energy for electron extraction from the solid surface (i.e., electron affinity for semiconductor, and WF for metal), compared to that of the bare surface without PEI. However, for double contacts at the interfaces of metal/PEI/semiconductor (Figure S13c, Supporting Information), this “‘pushing back”’ may act at both contacts, and the location of the counter charge on either side of the interface inverts the net dipole direction. As the Al is more chemically “softer” (polarizable) than the *n*‐Si,^[^ ^67^ ^]^ the negative charge balancing the positive amines in PEI is preferably located on the metallic side rather than on the semiconductor side, thus rearranging the net dipole pointing from Al towards *n*‐Si.

The effect of this PEI dipole layer on the interface between *n*‐Si and Al was therefore investigated by ultraviolet photoelectron spectroscopy (UPS). Noting that the thickness of the Al over‐layer was controlled to be ultrathin to let the buried interfaces accessible for measurements. As shown in Figure 4d, the WF of *n*‐Si/ill‐PEI/Al contact, determined by linear extrapolation of the photoemission cut‐off, is decreased from 4.28 eV for *n*‐Si/Al to 3.34 eV, which is much lower than that of *n*‐Si/dark‐PEI/Al (3.49 eV), indicating the largest dipole induced by the high‐density amines in ill‐PEI. The introduction of amines between *n*‐Si and Al gives rise to the effectively raised Femi level of the interface (with a downshift *E*_VAC_ of *∆* as shown in Figure S13, Supporting Information). Therefore, a low WF value of the *n*‐Si/ill‐PEI/Al contact results a large downward BB at *n*‐Si surface, with the electrons accumulating at interface whereas the holes drifting to bulk. The resulted high concentration of surface electrons as well as the corresponding low concentration of surface holes are also responsible for the low *ρ*_c_ and high *τ*_eff_ as mentioned above. In addition, compared with dark‐PEI/Al, the ill‐PEI/Al contact suppresses the Si peak much more severely in the HOMO region, suggesting a better coverage of the ill‐PEI/Al on *n*‐Si, in congruence with the AFM results. This high‐quality contact coverage is also important for the tarp passivation at the interface. Although the *τ*_eff_ of the *n*‐Si/thick‐PEI/Al contact is smaller than that of the *n*‐Si/ill‐PEI/Al, we find that the WF of the *n*‐Si/thick‐PEI/Al (3.27 eV) is however lower than that of the *n*‐Si/ill‐PEI/Al. This difference can be explained by the asymmetric distribution of amines in adsorbed ill‐PEI interlayer,^[^ ^68^ ^]^ as the *τ*_eff_ improvement is dominant by the amines at *n*‐Si/ill‐PEI interface (right side of the PEI as shown in Figure S13c, Supporting Information) while the WF reduction is dominant by the amines at ill‐PEI/Al interface (left side of the PEI as shown in Figure S13c, Supporting Information). Compared with the spin‐coated thick‐PEI, in which the distribution of amines is symmetrical along the thickness direction, the amines in adsorbed ill‐PEI prefer to localize very close to the *n*‐Si/ill‐PEI interface being electrostatically attracted by the illuminated *n*‐Si surface during the light‐promoted adsorption, thus resulting an asymmetric distribution of amines in ill‐PEI with high‐density amines accumulating near the *n*‐Si/ill‐PEI side.

We further note that the RMS surface roughness (Figure S14, Supporting Information) of the Al over‐layer can be greatly reduced from 1.86 nm for *n*‐Si/Al and 0.65 nm for *n*‐Si/dark‐PEI/Al to 0.38 nm for *n*‐Si/ill‐PEI/Al, indicating a significantly improved contact quality between Al and *n*‐Si brought by ill‐PEI. It can be interpreted by the “nucleation‐induced seed layer” effect of the ill‐PEI, in which the high‐density amines provide a great number of reactive sites for anchoring and immobilization of the evaporated Al atoms.^[^ ^69^ ^]^ Thereby, because of this large‐area and intimate electronic contact between the metal conductor and the semiconductor surface, the derived regular and even interface is expected to be beneficial for the carries transport and thus a low *ρ*_c_. In addition, this smooth contact interface also benefits to mitigate the near‐infrared plasmonic absorption losses at the metal electrode, which will be more important for the application in tandem solar cells.^[^ ^70^ ^]^ The XPS measurements were performed to identify the chemical reaction between Al atoms and amines (Figure S15, Supporting Information). The BE of the Al 2p peak for *n*‐Si/Al is observed at 74.5 eV, which negatively shifts to a lower BE of about 74.1 eV for the *n*‐Si/ill‐PEI/Al. While the BE of N 1s peak positively increases from 400.0 for *n*‐Si/ill‐PEI to 400.4 eV for *n*‐Si/ill‐PEI/Al. These chemical shifts indicate that the N atoms in amines contribute lone pairs of electrons to the empty orbits of Al atoms to create coordinate covalent bonds at the interface, as the evidence for the strong charge transfer between ill‐PEI and Al.

### 2.4 PV Performance of *n*‐Si Solar Cells Based on Full‐Area Passivating Electron‐Selective ill‐PEI Contacts

**Figure 5:**
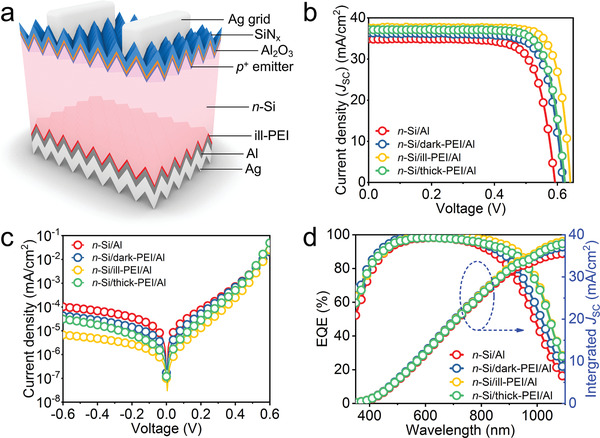
Performance of silicon solar cells with passivating electron‐selective ill‐PEI contact. a) Sketch of an *n*‐type silicon solar cell with a full‐area passivating electron‐selective ill‐PEI contact at the rear. b) Light and c) dark *J–V* curves of the cells with different rear contacts. d) External quantum efficiency and the integrated *J*_SC_ of the cells with different rear contacts.

Since the *n*‐Si/ill‐PEI/Al stack combines an efficient electron selectivity, an excellent trap passivation and a sufficiently low WF, its application as an electron contact is expected to yield high‐performance devices. To demonstrate its effectiveness at a device level, we fabricate a proof‐of‐concept silicon solar cell with a full‐area ill‐PEI/Al contact, a sketch of which is shown in **Figure** **5**a. The solar cells (2 cm × 2 cm) were fabricated on *n‐*Si wafers (1.0 Ω·cm) with a thickness of ≈175 *µ*m. The textured front side with random pyramids has a boron‐diffused *p* ^+^ emitter for hole collection, which was passivated by Al_2_O_3_/SiN*_x_* stack. The rear surface of cell is coated with a full‐area ill‐PEI/Al contact for electron collection. For comparison, a control cell with a direct *n*‐Si/Al rear contact was fabricated in the same batch. The *J*–*V* curves of the best‐performing *n*‐Si solar cells with different rear contacts (*n*‐Si/Al, *n*‐Si/dark‐PEI/Al, *n*‐Si/ill‐PEI/Al, and *n*‐Si/thick‐PEI/Al) under standard one‐sun illumination conditions are shown in Figure 5b, and the detailed PV parameters of cells are summarized in **Table** **1**. Since our devices adapt the same structure except for the rear contacts, all the variations in the PV parameters should be induced by the difference in the rear contacts. The control device with the direct *n*‐Si/Al contact exhibits a poor PCE of 15.5% with a relatively low *V*_OC_ of 0.592 V, a *J*_SC_ of 34.9 mA cm^−2^, and a FF of 75.2%, suggesting a severe recombination at the *n*‐Si/Al contact interface and a high Schottky barrier between *n‐*Si and Al. After introducing a dark‐PEI layer between *n‐*Si and Al, the PCE is enhanced to be 17.2%, with a *V*_OC_ of 0.617 V, a *J*_SC_ of 36.4 mA cm^−2^, and a *FF* of 77.0%, respectively, indicating an alleviated recombination and a reduced Schottky barrier of the *n‐*Si/dark‐PEI/Al contact. As expected, a further PCE improvement is achieved by replacing dark‐PEI with ill‐PEI. The device featuring the full‐area *n‐*Si/ill‐PEI/Al contact exhibits a champion PCE of 19.5%, associated with a *V*_OC_, *J*_SC_, and FF of 0.641 V, 37.6 mA cm^−2^, and 80.7%, respectively, representing an absolute efficiency gain of 4% over the control device. Compared with the conventical *n‐*Si/thick‐PEI/Al contact (with a PCE of 18.2%, a *V*_OC_ of 0.624 V, a *J*_SC_ of 37.1 mA cm^−2^, and a FF of 78.4%), the improved PV performance in *n‐*Si/ill‐PEI/Al based device can be directly attributed to the higher amine density and the lower film thickness of the homogeneous ill‐PEI monolayer, the combination of which can simultaneously reduce the contact resistivity (resulting in a higher FF) and the carrier recombination velocity (resulting in a higher *V*_OC_ as well as *J*_SC_) at the rear surface. Moreover, the statistical PCE distribution in Figure S16, Supporting Information also suggests good reproducibility of our ill‐PEI based devices, which can be attributed to the high‐quality contact due to the highly uniform and conformal coverage of the ill‐PEI even at the textured surface.

**Table 1:** The best performance of the silicon solar cells with a full area of *n*‐Si/Al, *n*‐Si/dark‐PEI/Al, *n*‐Si/ill‐PEI/Al, and *n*‐Si/thick‐PEI/Al rear contacts

Contact type	*V*_OC_ [V]	*J*_SC_ [mA cm^−2^]	FF [%]	PCE [%]	*R*_series_ [Ω cm]	*R*_shunt_ [kΩ cm]
*n*‐Si/Al	0.592	34.9	75.2	15.5	0.62	2.8
*n‐*Si/dark‐PEI/Al	0.617	36.4	77.0	17.2	0.40	2.1
*n*‐Si/ill‐PEI/Al	0.641	37.6	80.7	19.5	0.27	6.1
*n‐*Si/thick‐PEI/Al	0.624	37.1	78.4	18.2	0.36	6.0

The steady‐state *J*–*V* characteristics of the four types of devices in dark are shown in Figure 5c. All *J*–*V* curves exhibit rectifying characteristics, and the saturation current density is dramatically suppressed for the device with a *n‐*Si/ill‐PEI/Al contact. This suggests a reduction of the series resistance (*R*_s_), and an increase in the shunt resistance (*R*_sh_) for the *n‐*Si/ill‐PEI/Al based device, in good agreement with the improved cell parameters shown in Table 1. The corresponding external quantum efficiency (EQE) and integrated *J*_SC_ spectra are also shown in Figure 5d. All the devices show a high EQE in the short wavelength range (< 800 nm), which demonstrates a low reflection and carrier recombination at the front side because of the excellent antireflection and surface passivation by the Al_2_O_3_/SiN*_x_* stack. In contrast, the EQE in the long wavelength range (>800 nm) is poor for the control device, and is clearly improved by the insertion of the PEI interlayers. The best long‐wavelength response is observed for the device with a *n‐*Si/ill‐PEI/Al contact. This result confirms that the carrier recombination at the rear surface of the *n‐*Si is largely suppressed due to the strong field‐effect passivation induced by the high‐density Lewis‐base amines in ill‐PEI, consistent with the improvement in *V*_OC_ and with the reduction in saturation current density presented above. The calculated *J*_SC_ by integrating the product of EQE and air mass (AM) 1.5G spectrum for the *n‐*Si/ill‐PEI/Al based device is proven to be 38.5 mA cm^−2^, in good agreement with the measured *J*_SC_ value from *J*–*V* tests.

We then perform a comparison to state‐of‐the‐art electron‐selective contacts recently reported in the literature. Table S2, Supporting Information summarizes the performance parameters of the *n‐*Si based devices based on various full‐area electron‐selective contacts. Despite that the *J*_SC_ of our *n‐*Si/ill‐PEI/Al based device is limited by the unoptimized front grid (Figure S17, Supporting Information), the high values of *V*_OC_ and FF still make the PCE be considered one of the best results for devices with solution‐processed electron‐selective contacts, which is even higher than that of the silicon heterojunction solar cells with double‐layered a‐Si:H/PEI contact stacks. Moreover, it is of note that the ill‐PEI based single‐layered contact employed here has a solution‐processed, annealing‐free and low‐cost fabrication process, presenting a major advantage over the conventional phosphorus diffused and vacuum‐deposited contacts. In addition, this full‐area contact allows a low process complexity and a simple 1D current flow pattern at the rear side. These results indicate that the efficient electron selectivity, excellent surface passivation and low WF of the *n‐*Si/ill‐PEI/Al heterocontact can be maintained on the device.

In the work reported above, we considered the light‐promoted adsorption of Lewis base films on *n‐*Si substrates for passivating electron‐selective contacts. Alternatively, the PSS, one type of anionic polyelectrolytes with strong Lewis acids, would be another possible candidate as the adsorbate for passivating hole‐selective contacts. To illustrate the generality of the light‐promoted electrostatic adsorption, we followed similar procedures to adsorb PSS monolayers on *p‐*type Si (*p*‐Si) substrates. As shown in Figure S18, Supporting Information, a similar effect is observed that the PSS layer adsorbed under illumination (ill‐PSS) shows a clearly improvement in *τ*_eff_ from 181.4 *µ*s (for the control film adsorbed in dark condition, dark‐PSS) to 269.8 *µ*s, indicating a strong field‐effect passivation by the high‐density negative‐charged sulfonic acids in ill‐PSS, the adsorption of which can be promoted by the attraction of the photo‐generated nonequilibrium electrons on the *p‐*Si surface (Note S8, Supporting Information). Moreover, to determine whether the passivating carrier‐selective characteristic of the ill‐PEI based contact is ubiquitous, we further employed this strategy to the inverted PVK solar cells utilizing PCBM/ill‐PEI/Ag stacks as the electron‐selective contacts. As shown in Figure S19, Supporting Information, the BB across the interface of PVK and PCBM is cancelled by the illumination, and the photo‐generated nonequilibrium electrons contribute to the adsorption of the high‐density ill‐PEI monolayer (Note S9, Supporting Information). As a consequence, we observed a similar improvement of all PV parameters in device with structure of ITO/NiO*_x_*/PVK/PCBM/ill‐PEI/Ag compared to the device with dark‐PEI (Table S3, Supporting Information). These results suggest that there is likely to be a universal effect of illumination on the electrostatic adsorption of high‐density and ultra‐thin films from solution to semiconductor surfaces, independent on the charge type of adsorbate and the kind of semiconductor.

## 3 Conclusion

In summary, we developed a low‐temperature and solution‐processed light‐promoted adsorption method for the deposition of high‐density PEI monolayers as promising passivating electron‐selective contacts. The promoted adsorption is attributed to the significantly enhanced electrostatic interaction between the positive PEI segments and the negative semiconductor surface induced by the photo‐generated nonequilibrium carriers. The XPS, AFM, SKPM, and CA characterizations reveal that the light‐promoted adsorbed PEI monolayer exhibits a decreased film thickness but an increased amount of amines and an uniform surface coverage, as compared with the conventional PEI layer that deposited by spin‐coating and by dip‐coating without illumination. The derived high‐density amines in the angstrom‐scale PEI monolayer are demonstrated to simultaneously provide a low‐resistance electrical contact for electrons, a moderate field‐effect passivation to *n‐*Si surface, and an enhanced dipole formation at *n‐*Si/Al interface. As a result, the implementation of this high‐performance passivating electron‐selective contact on the rear side of an *n‐*Si solar cell leads to a PCE of 19.5% with a *V*_OC_ of 0.641 V, a *J*_SC_ of 37.6 mA cm^−2^ and an FF of 80.7%, which is one of the best results for devices with solution‐processed electron‐selective contacts. The present work demonstrates that the light‐promoted adsorption method can be used as a general way to realize efficient PCSCs not only for silicon‐based device platforms but also for diverse optoelectronic applications.

More broadly, we infer that this light‐promoted electrostatic adsorption method, which simply involved the illuminated substrates being contacted with a target solution, also provides a generic pathway for the deposition of highly uniform, smooth, dense, and ultra‐thin coatings. The advantages of this method are as follows: 1) This method can be anticipated in the unrestricted illumination. Not only the conventional (low‐energy) carriers generated in semiconductor, but the “hot” (high‐energy) carriers excited in metal can also take effect during the adsorption. 2) The substrate compatibility is manifold. Since the electrostatic adsorption enables preparation of nearly ideal conformal coatings on substrates with readily controlled thickness without being overly sensitive to surface topography, it can be widely applied to textured surfaces, spherical particles, inside pores, and onto other more complex geometries. 3) Independent of the target adsorbates. The concept can be extended to the deposition of various charged materials such as polymers, inorganic salts, organic molecules, DNA, colloids, nanoparticles, graphene oxide, biomolecules, lipids, and biological objects. Moreover, the charge fractions can be positive, negative or amphoteric and either inherent or foreign (grafted, photoinduced, or even frictional), and can be simply tuned by the solution concentration, PH, and ionic strength. 4) Patternable adsorption. In combination with simply optical masking techniques, the adsorption can be spatially modulated to directly photopattern the adsorbed layer. 5) The process control is simple and low‐cost. The use of the simple and fast dipping method does not need expensive precision instruments, and saves target solution which can be recycled and reused. 6) Potential for mass production. As a feasible approach for high‐throughput mass production, the process can be simply changed from a labor‐intensive technology to automation by utilizing mechanical immersion machines. Based on these advantages, this method can be generalized to other applications such as photodetectors, layer‐by‐layer self‐assembly, nanopattern and particle cleaning.

## Conflict of Interest

The authors declare no conflict of interest.

## Supporting information

Supporting Information
