# Supplementary material for: Light‐Promoted Electrostatic Adsorption of High‐Density Lewis Base Monolayers as Passivating Electron‐Selective Contacts
Source: Adv Sci (Weinh). 2021 Jan 4;8(5):2003245. doi: 10.1002/advs.202003245 (PMC7927610; doi:10.1002/advs.202003245)
Supplement: Supplementary file 1 — Supporting Information [file ADVS-8-2003245-s001.pdf]

© 2020 Wiley-VCH GmbH

ADVANCED SCIENCE
Open Access

Supporting Information

for *Adv. Sci.,* DOI: 10.1002/advs.202003245

Light-promoted Electrostatic Adsorption of High-density Lewis base Monolayers as Passivating Electron-selective Contacts

*Xi Yang, Zhiqin Ying, Zhenhai Yang, Jia-Ru Xu, Wei Wang, Jiajia Wang, Zenggui Wang, Lingze Yao, Baojie Yan, and Jichun Ye**

© 2020 Wiley-VCH GmbH

Supporting Information

Light-promoted electrostatic adsorption of high-density Lewis base monolayers as passivating electron-selective contacts

*Xi Yang, Zhiqin Ying, Zhenhai Yang, Jia-Ru Xu, Wei Wang, Jiajia Wang, Zenggui Wang, Lingze Yao, Baojie Yan, and Jichun Ye**

Experimental Section

Light-promoted adsorption of PEI: The PEI (M_W ~ 25,000 by LS, branched, Sigma-Aldrich) was diluted to 4 mg/ml by ethanol. The values of pH and ionic strength were not specially modified. One-side polished, *n*-type Czochralski (CZ) silicon wafers (1–3 Ω·cm) with (100) crystal orientation and 270 μm thickness were used as the adsorption substrates. Prior to the PEI adsorption, the samples were subjected to an optimized RCA cleaning process comprising an HF (1% in volume) dip to remove the oxide layer, an etch in 25% tetramethylammonium hydroxide (TMAH) for 10 min at ~ 80 °C to remove saw damage, an HF (1% in volume) dip to remove the native oxide layer, an RCA-1 (NH_3H_2O:H_2O_2:DI water = 1:1:5) at 65 °C for 10 min to remove organic residues, an HF (1%) dip to remove the native oxide layer, an RCA-2 (HCl:H_2O_2:DI water = 1:1:5) at 65 °C for 10 min to remove the metal ions and inorganic residues, an HF (1% in volume) dip to remove the native oxide layer and finally a pulled drying from the HF solution. Immediately, these samples were immersed into the PEI solution for 1 min. For ill-PEI samples, a white-light LED with tunable intensity in a range of 1,000–5,000 lux was used for irradiation to manipulate the PEI adsorption. For dark-PEI samples, the adsorption was proceeded without illumination (with the illuminance blew 80 lux). After the adsorption, the samples were thoroughly rinsed twice with plenty of DI water to remove the weakly adsorbed PEI chains and then dried in nitrogen flow. It is noteworthy that, without the thorough washing, these weakly adsorbed PEI chains would significantly increase the film

thickness and the surface roughness of the adsorbed film, yielding a poor film quality. For thick-PEI samples the PEI solution was spin-coated onto the silicon substrate at 5,000 r.p.m. and then dried in nitrogen flow.

PEI Characterization: The chemical composition of the adsorbed PEI was determined by the high-resolution XPS (Kratos Axis Ultra Ltd.) equipped with a monochromatic Al Kα X-ray source (1486.6 eV) and a hemispherical analyzer in ultrahigh vacuum with a base pressure of 1×10^{-10} mbar. The survey and high-resolution spectra were performed at fixed analyzer pass energy and quantified using empirically derived relative sensitivity factor provided by the manufacturer. The binding energies were calibrated by the C 1s peak, which were set at 284.6 eV, and Gaussian line shapes were used for these fits. The data were analyzed using a commercially available software of CasaXPS. The thickness of the adsorbed PEI layer was obtained by the spectroscopic ellipsometry measurement (M-2000). The localized changes of the contact potential of the *n*-Si surface (with and without illumination, before and after PEI adsorption) was determined by the SKPM measurements (Veeco Dimension-3100V). During SKPM measurements the sample was grounded. An Au substrate was used as reference to monitor any tip wear after multiple measurements. Amplitude modulation SKPM was used to obtain a high signal-to-noise ratio as opposed to that of frequency modulation. All SKPM measurements were performed in dual pass mode to completely eliminate the topography effect. When the tip with an applied bias scans the surface of the sample, the CPD created between the tip and the sample surface was used to infer the spatial variation of surface potential and the homogeneity of adsorbed layers. The same white-light LED used for light-promoted PEI adsorption was utilized as the applied illumination to induce the surface photovoltage during the SKPM measurements. The surface morphologies of the samples were obtained by the AFM measurements (Veeco Dimension-3100V). The CA was determined by CA tester (DataPhysics Instruments GmbH). The electron-selective property of the PEI based contact was determined by making Schottky structures on *p*- and *n*- Si substrates with

different PEI interlayers. The top side of the structure was fabricated by first depositing a full-area PEI layer, followed by a series of Al circular contacts of different diameters (Fig. S8), using thermal evaporation with a shadow mask. The back side ohmic contact featured a full-area gallium-selenide eutectic and gallium-indium eutectic (99.99% Sigma-Aldrich) for *p*- and *n*- Si, respectively. Then the *J–V* measurements were carried out at room temperature using a semiconductor parameter analyzer (Keithley 4200). The contact resistivity was extracted by the Cox and Strack method using a spreading resistance model. The passivation quality of the PEI based contact was assessed by using the QSSPC measurements. A symmetrical test structure (Fig. S9) was prepared on a double-side polished float-zone *n*-Si to allow the effective lifetime measurement with a Sinton lifetime tester (WCT-120). The measurements were made on quarter wafers which were sufficiently large to avoid strong edge recombination effects. The PEI film was deposited on both sides of the silicon substrate. An ultra-thin Al (~ 7 nm) capping layer was evaporated onto the surface to mimic a device contact, whilst still allowing sufficient light transmission for the injection dependent carrier lifetime to be measured by the photoconductance decay technique. The WF of the PEI based contact was determined by UPS (Krato Axis Ultra Ltd.) using a He-I excitation (21.22 eV). An Au reference WF at 5.1 eV was measured in the same measurement session, confirming the measurements accuracy.

Device fabrication and characterization: The proof-of-concept cells were fabricated on CZ *n*-type c-Si wafers with a resistivity of 2.0 Ω·cm and a thickness of 180 μm. The as-cut (2 cm × 2 cm) (100)-oriented silicon wafers were subjected to an alkaline solution of TMAH, DI water, isopropyl alcohol, and dissolved silicon at a temperature of 85 °C for 60 min, forming textured morphologies with an array of random pyramids. After the RCA cleaning procedure, a full-area boron diffusion was performed in a dedicated clean quartz furnace. The front textured surfaces were then passivated with a stack of 20 nm atomic layer deposited Al_2O_3 and 65 nm plasma enhanced chemical vapor deposited SiN_x. Note that the stack also functions

as an antireflection coating. The undiffused rear silicon surfaces were then immersed into the PEI solution to process the light-promoted adsorption as mentioned above. For thick-PEI based samples, the PEI solution were spin-coated onto the rear silicon surfaces at 5,000 r.p.m. and then dried in nitrogen flow. Finally, the samples were thermally evaporated the Al/Ag (80/800 nm) stacks as rear electrodes. A control cell with the direct contact of *n*-Si with Al at rear surface was prepared at the same time for comparison. The *J*–*V* behavior was measured under standard one sun conditions (100 mW/cm^2, AM1.5 spectrum, 25 °C) using a solar simulator (Oriel, Sol3A), which was calibrated with a certified Fraunhofer CalLab reference cell. The EQE spectral response measurements was conducted with a photo-modulation spectroscopic setup (Newport monochromator). The illumination intensity of solar simulator was calibrated using a standard Si reference cell with known spectral response.

Supplementary Note 1:

The amount of adsorbed PEI in the electrical double layer (EDL) is determined by the total potential energy (V_t) of the mutual interaction between the PEI segments and *n*-Si (i.e., the adsorption can be proceeded and promoted if the total interaction energy of the system is negative in sign and sufficiently large, and vice versa), which is contributed by the long-range van der Waals (V_a), the electrostatic double layer potentials (V_e), and the electrostatic repulsion (V_r) between adsorbed molecules and molecules approaching the *n*-Si surface ($V_t = V_a + V_e + V_r$).

The V_a can be estimated using the approximate formula for retarded interaction given by Gregory:[1]

$$V_a(x) = -\frac{AR}{6x}\left(\frac{1}{1 + 14\frac{x}{\lambda}}\right) \tag{1}$$

where A is the Hamaker constant, R is the radius of the charged PEI segment, x is the distance of separation between the PEI and the silicon surface, and λ is a characteristic wavelength for the interaction (typically in the range of 90 to 100 nm). Thus, the V_a is attractive (negative) in nature for most situations.

The V_e can be estimated using the analytical expression provided by Hogg:[2]

$$V_e(x) = \pi\varepsilon_0\varepsilon_r R\left(\zeta_1^2+\zeta_2^2\right)\left\{\frac{2\zeta_1\zeta_2}{\zeta_1^2+\zeta_2^2}\ln\left[\frac{1+e^{-kx}}{1-e^{-kx}}\right]+\ln\left[1\text{-}e^{-2kx}\right]\right\} \quad (2)$$

where the ε_0 is the permittivity under vacuum, ε_r is the relative dielectric permittivity of the intervening medium, κ is the reciprocal of the Debye length, ζ_1 is the surface potential of the semiconductor, and ζ_2 is the zeta-potential of the PEI solution. As the signs of ζ_1 and ζ_2 are opposite (here negative for *n*-Si surface whereas positive for PEI), the V_e is also attractive (negative) in this system.

However, considering the usual case of identical charge between PEI monomers (i.e., $\zeta_1 = \zeta_2 = \zeta_0$), the V_r is repulsive (positive) and can also be evaluated analytically giving:

$$V_r(x) = \frac{\varepsilon_r R\zeta_0^2}{2}\ln\left[1+e^{-kx}\right] \quad (3)$$

As can be seen by analyzing the equations (1-3), the total driving force in the adsorption process is determined by the balance between electrostatic attraction of charged PEI segments to the *n*-Si ($W_{att} = V_a + V_e$), which favors a linear flat conformation with chains situating in trains, and the repulsion in PEI segment-segment ($W_{rep} = V_r$), which favors a coiled conformation with loops and tails pointing away from the substrates.[3] It is worth noting that as soon as the PEI adsorbed onto the *n*-Si surface, both V_a and V_e in W_{att} approach maximum when the x is approaching 0, thus these adsorbed PEI segments are tightly-attached on the plane parallel to the surface of the substrate and can be hardly washed away. While for the PEI segments distributed along the direction perpendicular to the surface, where x is larger than R, the V_r in W_{rep} between these PEI segments is increased with the x and gradually

cancels out the W_{att}, thus resulting loosely-attached PEI segments which can be easily washed away.

Supplementary Note 2:

Under dark condition, the Fermi level (E_F) of the bulk *n*-Si occurs at a higher energy than that of the surface trap states (E_{SS}) which have a single and discrete energy level within the bandgap. In order to achieve electronic equilibrium between the surface and the bulk, some electrons are transferred from bulk to surface and are then trapped by E_{SS} in the surface space-charge region (SCR). These charges give rise to an electrostatic potential drop across the SCR and an upward band bending (BB) of the vacuum level (E_{VAC}) at the surface. As the E_F of bulk finally aligns with the energy level of the E_{SS}, the energy distance between the E_F and the conduction band (E_C) is increased at the surface (behavior which has been termed Fermi level pinning), thereby resulting a built-in field pointing outward from bulk to surface. Such a built-in field repels the majority-carriers (electrons) from the surface to the bulk, leaving behind a positive-charged depletion layer full of immobile ionized donors.

Differently, under illumination, the photogenerated electron–hole pairs are created due to band-to-band (or trap-to-band) transition in semiconductor. The electron and hole density in *n*-Si are no longer determined by the same E_F. It is useful to define quasi-Fermi levels, $E_{F,n}$, and $E_{F,p}$, one for electrons and another for holes, as given by:[4]

$$E_{F,n} = E_C - \ln\left(\frac{N_C}{n_0+\Delta n}\right) \quad (4)$$

$$E_{F,p} = E_V + \ln\left(\frac{N_V}{p_0+\Delta p}\right) \quad (5)$$

where N_C and N_V are the density of energy states around the conduction band and valence band, respectively, n_0 and p_0 is the number of equilibrium electrons and holes under dark

conditions, respectively, Δn an Δp is the photo-generated nonequilibrium electrons and holes (usually, $\Delta n = \Delta p$), respectively. Given that the $\Delta n \ll n_0$ and $\Delta p \gg p_0$ in *n*-Si, the $E_{F,n}$ remains unchanged (i.e., $E_{F,n} = E_F$), whereas the $E_{F,p}$ is shifted considerably downwards to align with the E_{SS}, thus resulting a decreased band bending.

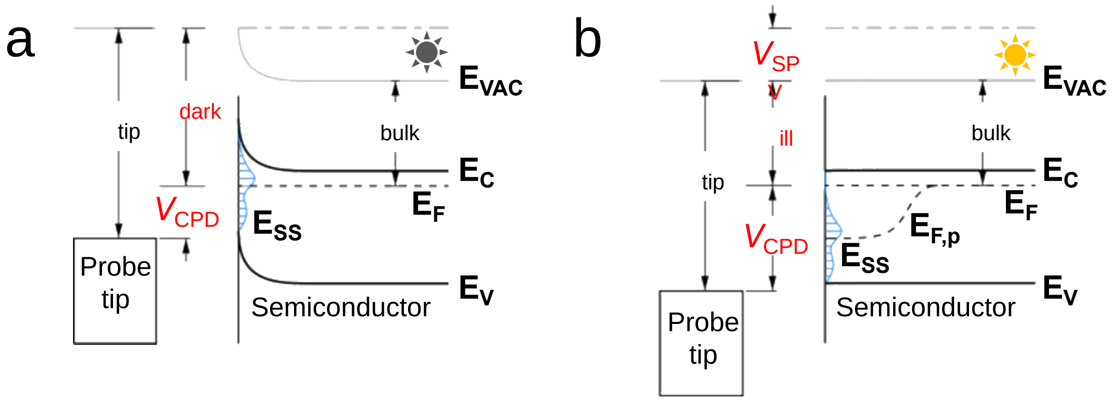

Figure S1. Energy diagrams of the *n*-Si and probe tip under the SKPM measurements (a) without and (b) with illumination. φ_{tip} and φ_{bulk} is the potential of the probe tip and bulk Si, respectively. φ_{dark} and φ_{ill} is the surface potential of the Si under dark and under illumination, respectively. V_{CPD} is the contact potential difference measured from the SKPM. V_{SPV} is the surface photovoltage of the *n*-Si induced by illumination.

Supplementary Note 3:

The surface potential of the *n*-Si ($\varphi_{surface}$) can be given by

$$\varphi_{surface} = \varphi_{tip} - V_{CPD} \tag{6}$$

$$V_{SPV} = \varphi_{dark} - \varphi_{ill} = \left(\varphi_{tip} - V_{CPD\text{-}dark}\right) - \left(\varphi_{tip} - V_{CPD\text{-}ill}\right) = V_{CPD\text{-}ill} - V_{CPD\text{-}dark} \tag{7}$$

where φ_{tip} is the surface potential of the conductive tip, $V_{CPD\text{-}dark}$ and $V_{CPD\text{-}ill}$ is the measured V_{CPD} under dark and under illumination, respectively, thus, the increased V_{CPD} corresponds to the positive V_{SPV} as well as the down-ward BB. Under illumination, driven by the built-in field in SCR, the photo-generated holes and electrons drift to the surface and the bulk,

respectively, thus resulting a decreased $\varphi_{surface}$ and BB. After switching off the illumination, the carrier concentration in bulk quickly returns to its original steady-state values, and the hole concentration at surface starts to decrease as a result of back diffusion and recombination.[5] Therefore, both the $\varphi_{surface}$ and the BB recover toward their dark equilibrium values.

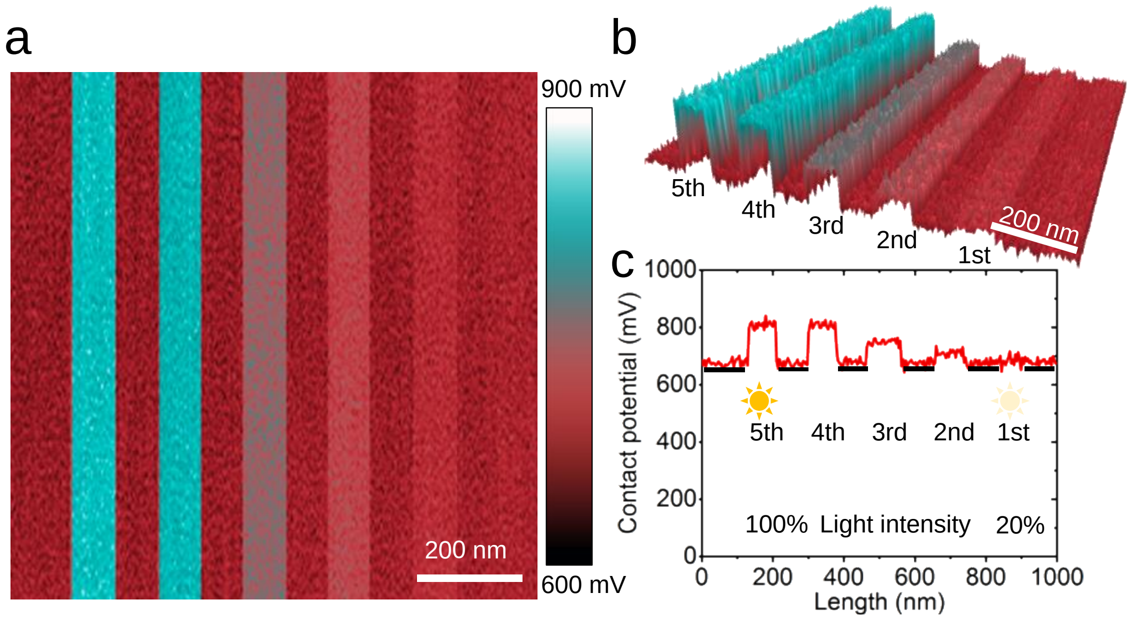

Figure S2. The surface potential (a) two-dimensional and (b) three-dimensional map of *n*-Si under different illuminance. (c) The surface potential profiles extracted from the SKPM map, which has five step-like features (first to fifth), with the fifth step being the highest surface potential shift (left most) under 5000 lux. A clear increasing trend in V_{CPD} is seen as the illuminance is increased from 0 to 5000 lux, attesting to the decreasing BB at *n*-Si surface with increasing number of photo-generated electron–hole pairs. With further increase in illuminance, the V_{CPD} changes rather insignificantly. Under this photosaturation condition, the redundant generated electron–hole pairs lost by back diffusion and recombination in the bulk, resulting an ultimate flat BB on the *n*-Si surface.

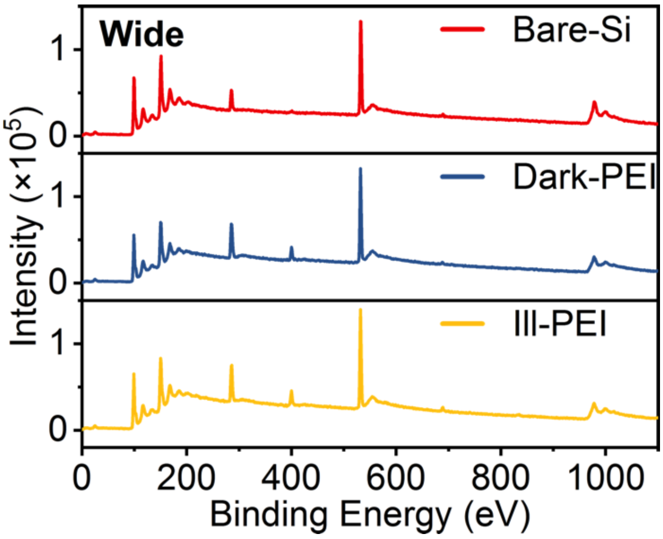

Figure S3. The XPS survey spectra of Si 2p core level of bare Si, dark-PEI/Si and ill-PEI/Si.

Table S1. The surface compositions (atom%) of elements from XPS results for bare-Si, dark-PEI and ill-PEI samples.

	**C 1s**	**N 1s**	**O 1s**	**Si 2p**	**C−N/C−C**	**N^+/N**
Bare-Si	17.6	N/A	29.3	53.1	N/A	N/A
Dark-PEI	31.9	5.1	24.5	38.5	2.2	10.9
Ill-PEI	28.0	6.7	23.1	42.2	3.1	15.4

Supplementary Note 4:

The deconvoluted C1s region in the XPS spectra of bare-Si shows three different components: the first peak located at the binding energy (BE) of 284.6 eV is attributed to the hydrocarbons (C−H) and aromatic carbons (C−C), which is used as reference peak corresponds to the rest of

the predominant aliphatic chain, the second and third peaks located at the BE of 286.5 eV and 288.2 eV are related to the carbon bound to oxygen of C−O and C=O, respectively. After the PEI adsorption, the C 1s XPS bands broaden to the high-energy side and a fourth carbon component assigned to the C−N bonds in the PEI molecule is observed at the BE of 285.6 eV, while the other three carbon components are evident at the same BE.

The similar changes are also observed in the N 1s region. No N 1s signal is present in bare Si sample. After the PEI adsorption, a strong asymmetric peak, which can be resolved into two components for the N atoms, appears in both dark- and ill-PEI samples, indicating the partial ionization of PEI due to its weak polyelectrolyte nature.[6] The deconvoluted peak centered at low BE of 400.0 eV is assigned to the N groups in the neutral amines, whereas the other one at high BE of 402.1 eV is associated with the N^+ groups in the protonated amines.[7]

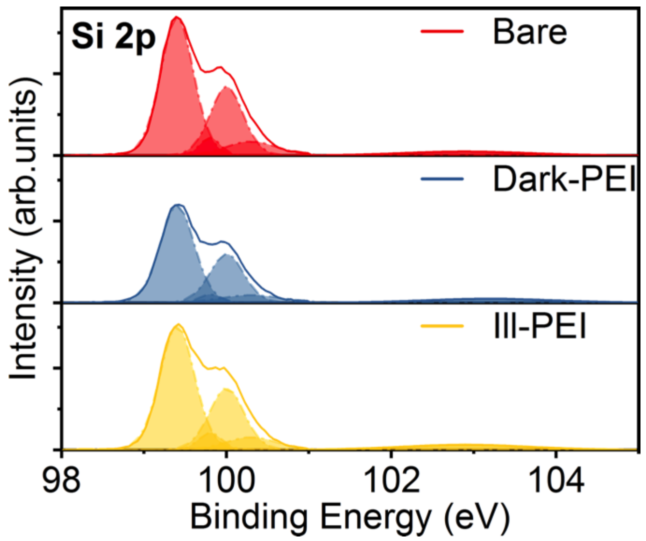

Figure S4. The high-resolution XPS spectra of Si 2p core level of bare Si, dark-PEI/Si and ill-PEI/Si.

Supplementary Note 5:

To quantitate the influence of the illumination on the thickness of the adsorbed PEI layer on *n*-Si surface, the fine XPS spectra for Si 2p are examined in detail. In the deconvoluted Si 2p narrow scan (Figure S4), the doublet peaks at 99.5 and 100.1 eV corresponds to the Si $2p_{3/2}$ and $2p_{1/2}$ levels of the *n*-Si substrate, respectively, while a nearly flat baseline within 103−104 eV indicates an oxide-free Si surface. The attenuation of the Si $2p_{3/2}$ signal of *n*-Si substrate can be used to infer the thickness of the adsorbed PEI monolayer by:[8]

$$d_{ML} = -\lambda_{ML}^{Si}\cos(\theta)\ln\left(\frac{I}{I_0}\right) \quad (8)$$

where d_{ML} is thickness of the adsorbed monolayer, I_0 and I are the Si 2p intensity before and after PEI adsorption, respectively, θ is the takeoff angle (from the surface normal, $\theta = 0^{\circ}$), and λ_{ML}^{Si} is the mean free path of the Si 2p photoelectron in the organic monolayers, taken to be 39.5 Å.[9] Inserting the corresponding Si $2p_{3/2}$ peak area values into the equation 8, the obtained values for the thickness of adsorbed PEI with and without illumination are 9.1 and 12.7 Å, respectively,

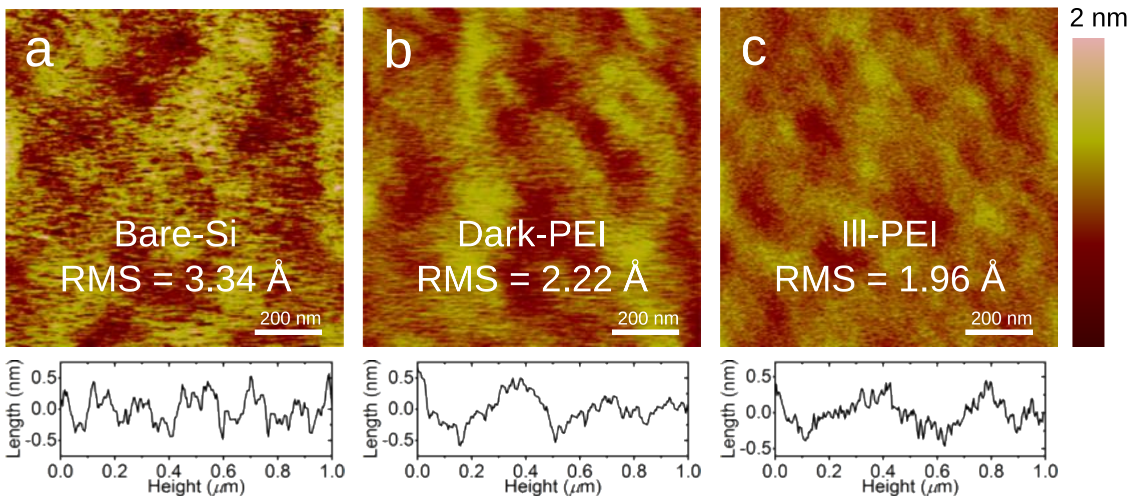

Figure S5. The topographical atomic force microscopy images (top) and the corresponding profilometric height line scans (bottom) for the surface of (a) bare-Si, (b) dark-PEI and (c) ill-PEI.

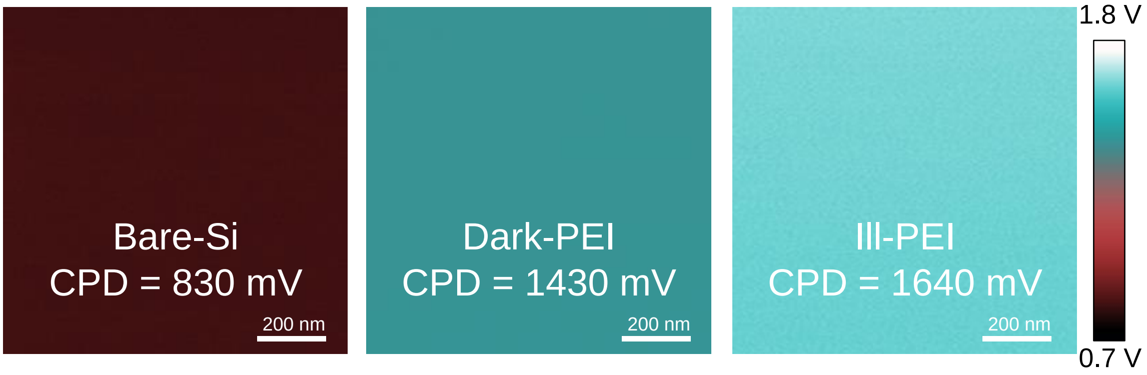

Figure S6. The SKPM images for the surface of (a) bare-Si, (b) dark-PEI and (c) ill-PEI.

Supplementary Note 6:

With a simple parallel plate capacitor model, the change in the surface potential (ΔV_{CPD}) after PEI adsorption can be estimated via the Helmholtz equation:[10]

$$\Delta V_{\mathrm{CPD}} = \frac{N\mu(\cos\theta)}{\varepsilon\varepsilon_0} \tag{9}$$

where N is the dipole density in PEI layer, μ is the dipole moment, which has an average title θ from the normal, ε is the relative permittivity of the PEI layer, and ε_0 is the permittivity of vacuum. The linear dependency of ΔV_{CPD} on N indicates that the density of amines in PEI layer determines the intensity of the electrostatic dipole and correlates linearly with changes in the potential.[11]

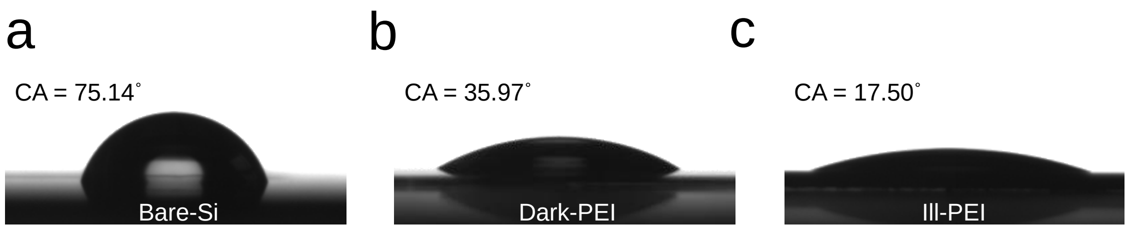

Figure S7. The contact angle images of deionized water drop on the surface of (a) bare-Si, (b) dark-PEI and (c) ill-PEI.

Supplementary Note 7:

As the hydrophilic nature of the polar amines in PEI,[12] the surface energy can also be used to probe the influence of illumination on the PEI density by measuring the contact angle (CA) of water droplet. We compare the CA of the respective three samples and find that both the dark-PEI and ill-PEI show the reductions in their CAs reaching 35.97˚ and 17.50˚, respectively, as compared with 75.14˚ for the bare-Si, confirming the successful adsorption of hydrophilic amines. This CA difference correlates with the change in the density of adsorbed amines, giving the largest value for bare-Si due to its hydrogen-terminated surface and lowest value for ill-PEI due to its dense amines and flat chains conformation.

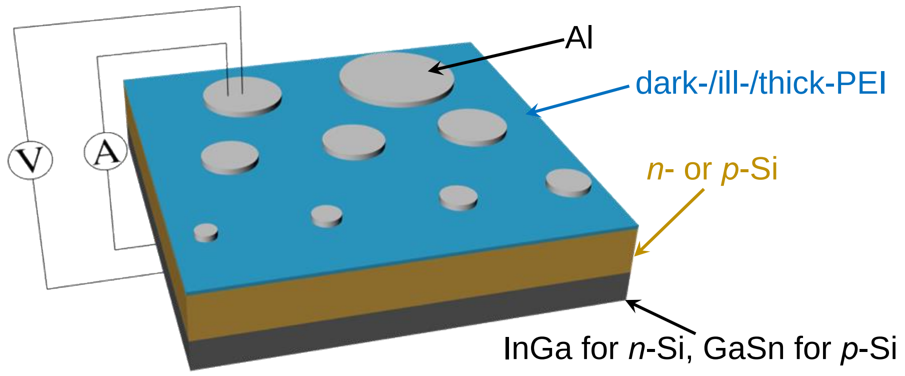

Figure S8. The schematic of the contact resistivity test structure. The contact resistivity can be extracted by the Cox and Strack method,[13] which involves a series of resistance measurements on the probe station with these different diameter front Al contacts. The GaSn and InGa eutectic are used to achieve the ohmic rear contacts to *p*-Si and *n*-Si, respectively.

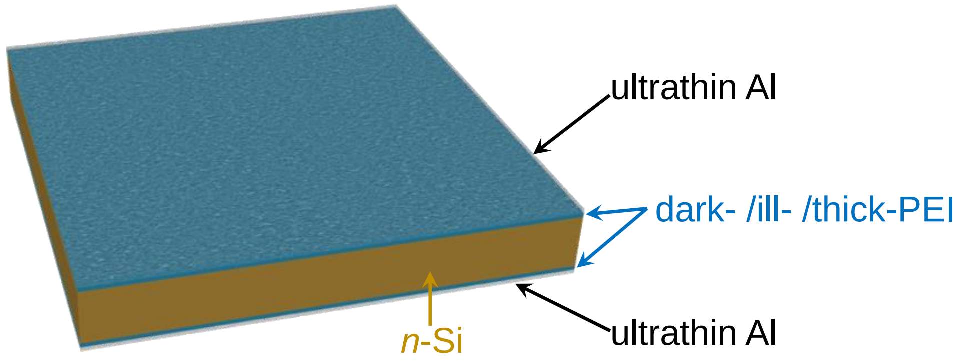

Figure S9. The schematic of the carrier lifetime test structure. The thickness of the ultrathin Al over-layer is controlled to be thin (<10 nm) to mimic a contact, while still transmitting sufficient light for the photo-conductance measurement.

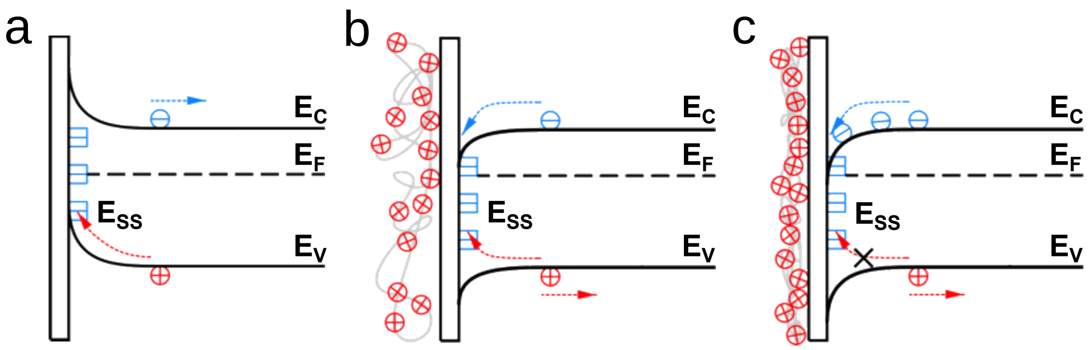

Figure S10. The illustration of the passivation mechanism for (a) *n*-Si/Al, (b) *n*-Si/dark-PEI/Al, and (c) *n*-Si/ill-PEI/Al contacts

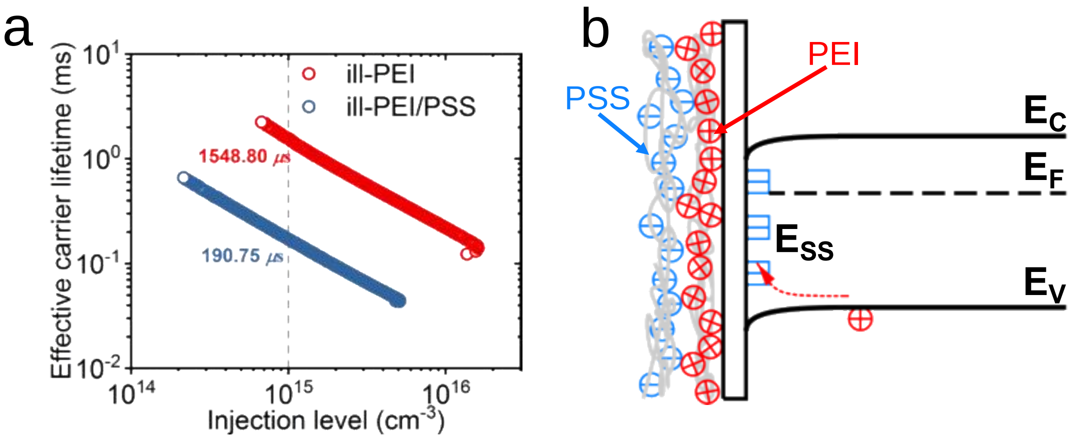

Figure S11. (a) Value of the effective carrier lifetime versus injection level measured for *n*-Si/ill-PEI/Al and *n*-Si/ill-PEI/PSS/Al contacts. (b) The illustration of the passivation mechanism for *n*-Si/ill-PEI/PSS/Al contact.

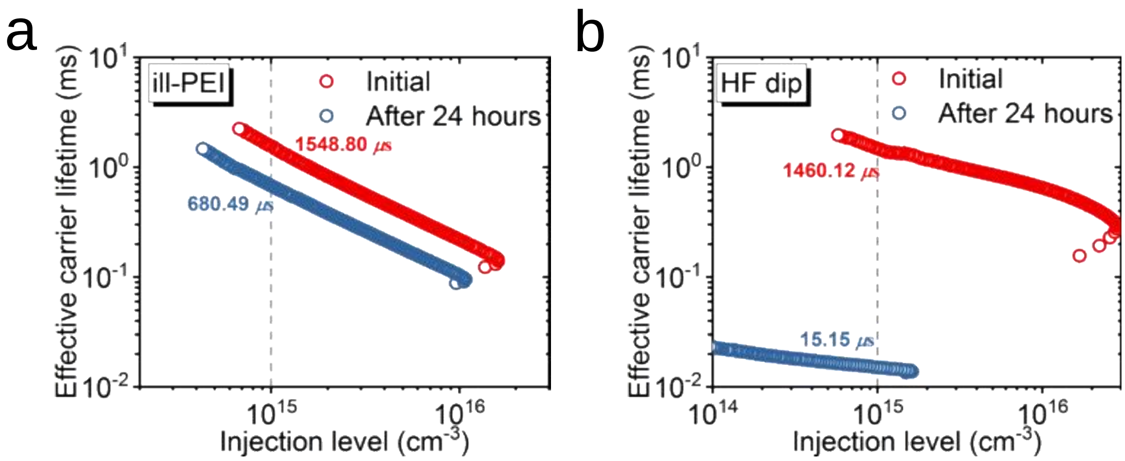

Figure S12. Value of the effective carrier lifetime versus injection level measured for (a) *n*-Si/ill-PEI/Al and (b) HF treated *n*-Si before (J_0 = 214 fA/cm^2) and after (J_0 = 6130 fA/cm^2) 24 hours air-exposure.

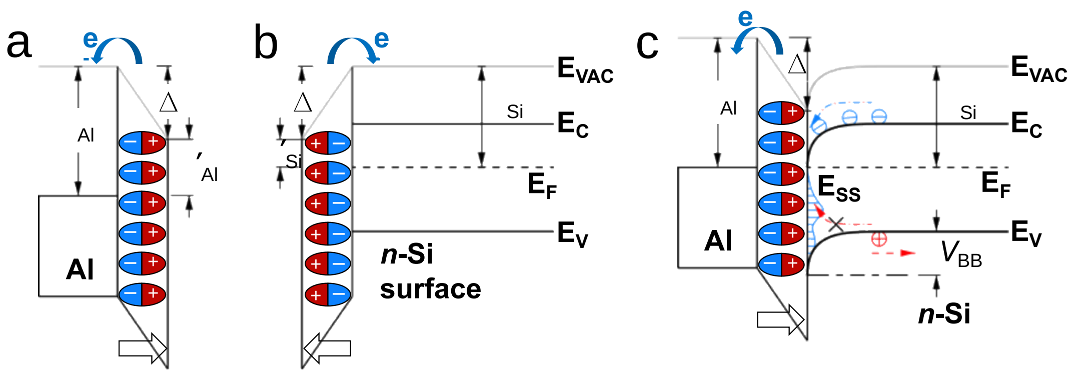

Figure S13. The energy level alignment diagrams near the single contact interfaces of (a) ill-PEI/Al and (b) ill-PEI/*n*-Si, and the double contact interfaces of (c) *n*-Si/ill-PEI/Al.

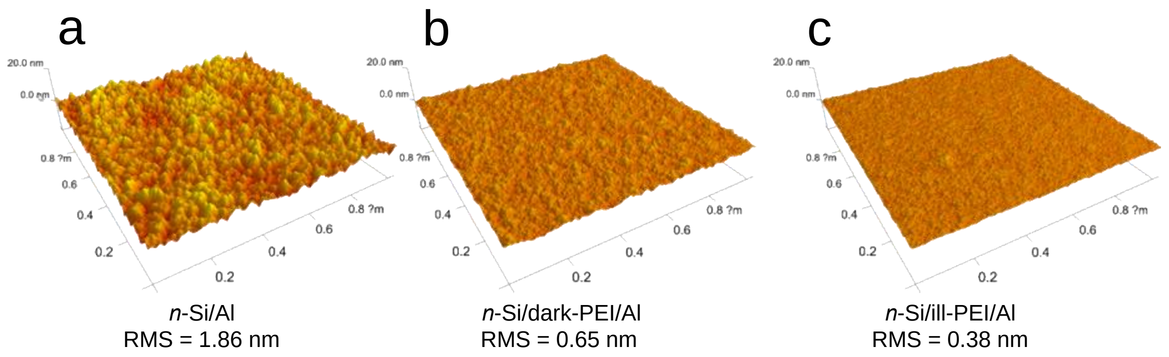

Figure S14. The three-dimensional atomic force microscopy images of the surface morphology of (a) *n*-Si/Al, (b) *n*-Si/dark-PEI/Al, and (c) *n*-Si/ill-PEI/Al. The scanning area is 1 μm × 1 μm.

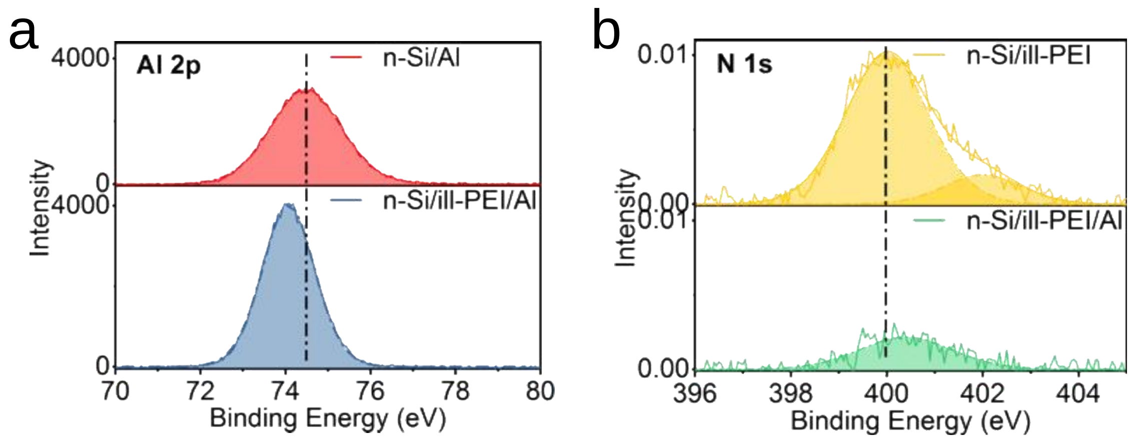

Figure S15. The high-resolution XPS spectra of (a) Al 2p core level of *n*-Si/Al contact without (top) and with (bottom) ill-PEI interlayer, and (b) N 1s core level of *n*-Si/ill-PEI contact without (top) and with (bottom) Al capping layer.

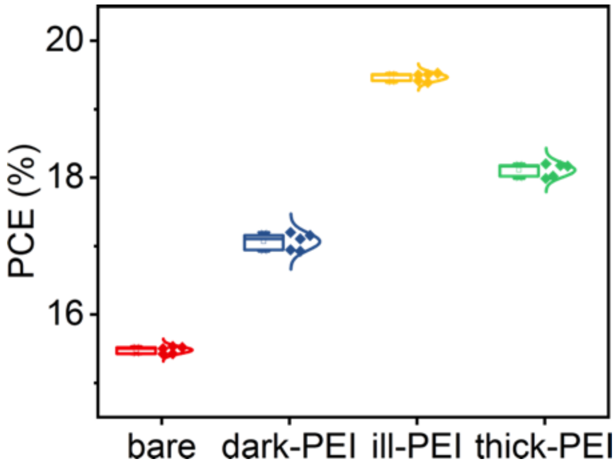

Figure S16. Statistical PCE distribution for n-Si cell with different full-area rear contacts.

Table S2. Comparison to the state-of-the-art electron selective contacts for silicon solar cells.[14-26]

	Contact materials	*V*oc [mV]	*J*sc [mA/cm^2]	*FF* [%]	PCE [%]	Method	Performance	Ref.
Multi-layered	a-Si:H/MgF_x	687	37.8	77.3	20.1	400 ˚C PECVD for a-Si:H<br>thermal evaporation for MgF_2	[a)]76 mΩ·cm^2<br>[b)]4088 µs; 10 fA/cm^2<br>[c)]3.5 eV	ACS Appl. Mater. Interfaces, 2016, 8, 14671-14677. [14]
	a-Si:H/Mg	636	38.0	78.4	19.0	400 ˚C PECVD for a-Si:H<br>thermal evaporation for Mg	310 mΩ·cm^2<br>N/A<br>3.7 eV	Appl. Phys. Lett., 2016, 109, 113901. [15]
	SiO_x/TiO_x	676	39.6	80.7	21.6	75˚C ALD for TiO_x<br>700 ˚C thermal oxidation for SiO_x<br>350˚C forming gas atmosphere annealing	20 mΩ·cm^2<br>650 µs<br>N/A	Adv. Mater., 2016, 28, 5891-5897. [16]
	SiO_x/TiO_x	674	39.8	82.5	22.1			Prog. Photovolt. Res. Appl., 2017, 25, 896-904. [17]
	SiO_x/poly-Si (TOPcon)	724	42.5	83.3	25.7	wet-chemical (HNO_3) for SiO_x<br>PECVD for phosphorous-doped Si<br>800˚C annealing post-treatment	50 mΩ·cm^2<br>4 fA/cm^2<br>N/A	Sol. Energy Mat er. Sol. Cells 2017, 173, 96.[18]
	a-Si:H/TiO_x/Ca	711	35.1	72.9	18.2	< 200˚C PECVD for a-Si:H<br>200˚C ALD for TiO_x<br>thermal evaporation for Ca	15 mΩ·cm^2<br>13.4 fA/cm^2<br>2.9 eV	Prog. Photovolt. Res. Appl., 2018, 26, 835-845. [19]
	TaO_x/Mg	638	37.8	79.3	19.1	250 ˚C ALD for TaO_x<br>thermal evaporation for Mg<br>hydrogenation post-treatment	350 mΩ·cm^2<br>22.5 fA/cm^2<br>3.27 eV	ACS Energy Lett., 2018, 3, 125-131. [20]
	SiO_x/TiN	644	37.9	81.9	20.0	UV-O_3 for SiO_x<br>sputtering for TiN	16.4 mΩ·cm^2<br>500 fA/cm^2<br>4.3 eV	Joule, 2019, 3, 1314-1327. [21]
	a-Si:H/b-PEI	720	37.0	72.9	19.4	200˚C PECVD for a-Si:H	156 mΩ·cm^2<br>2.4 ms<br>2.93 eV	ACS Energy Lett., 2020, 5, 897-902. [22]
Single-layered	MgO_x	629	39.5	80.6	20.0	thermal evaporation	17.5 mΩ·cm^2<br>950 fA/cm^2<br>4.1 eV	Adv. Energy Mater., 2017, 7, 1601863. [23]
	L-histidine	625	36.8	76.3	17.5	solution-processed<br>110 ˚C annealing	N/A<br>175 µs<br>N/A	J. Appl. Phys., 2018, 123, 024505. [24]
	Carbonates	624	38.89	79.9	19.4	thermal evaporation	90 mΩ·cm^2<br>N/A<br>2.23 eV	Adv. Energy Mater., 2018, 8, 1800743. [25]
	TaN	632	38.8	81.8	20.1	250 ˚C ALD	54 mΩ·cm^2<br>500-800 fA<br>4.3 eV	Adv. Energy Mater., 2018, 8, 1800608. [26]
	TiN	619	37.5	80.3	18.6	sputtering	165 mΩ·cm^2<br>1500 fA/cm^2<br>4.3 eV	Joule, 2019, 3, 1314-1327. [21]
	**ill-PEI**	**641**	**37.6**	**80.7**	**19.5**	**solution-processed<br>annealing-free**	**26.7 mΩ·cm^2<br>1549 µs; 387 fA/cm^2<br>3.34 eV**	**This work**

[a)] The contact resistivity of the contact.
[b)] The lifetime or/and the recombination current density of the contact.
[c)] The work function of the contact interface.

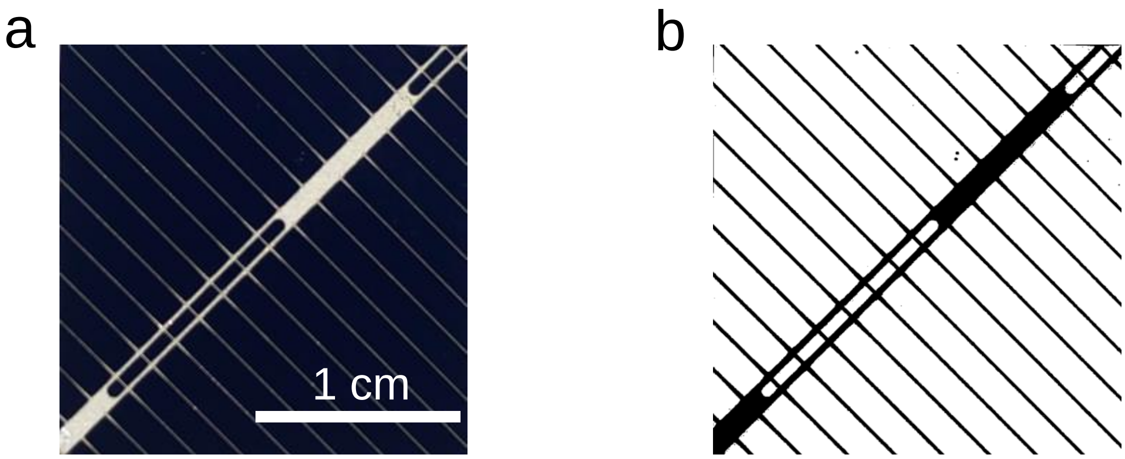

Figure S17. (a) Photo of the fabricated silicon solar cell. (b) The corresponding image for the shadowing losses of the metal grid processed by the image analysis software Image J.[27]

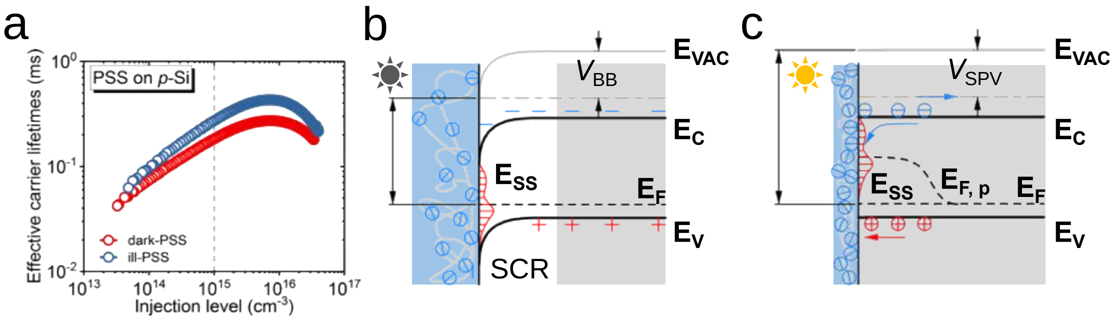

Figure S18. (a) Value of the effective carrier lifetime versus injection level measured for the PSS adsorbed on *p*-Si substrates (2-4 Ω·cm) without (dark-PSS) and with (ill-PSS) illumination. Band diagram of the *p*-Si in equilibrium with the PSS solution (b) in dark condition and (c) under illumination.

Supplementary Note 8:

During the dark-PSS adsorption, the V_{BB} for the movement of holes from bulk to surface is large, and the positive charges at surface trap states are compensated by the negatively ionized donors in SCR, thus hindering the PSS adsorption. While for the ill-PSS adsorption, the V_{BB} is compensated by the V_{SPV}, and the photogenerated holes are easily drawn towards the surface due to the eliminated potential barrier, thus resulting a promoted PSS adsorption.

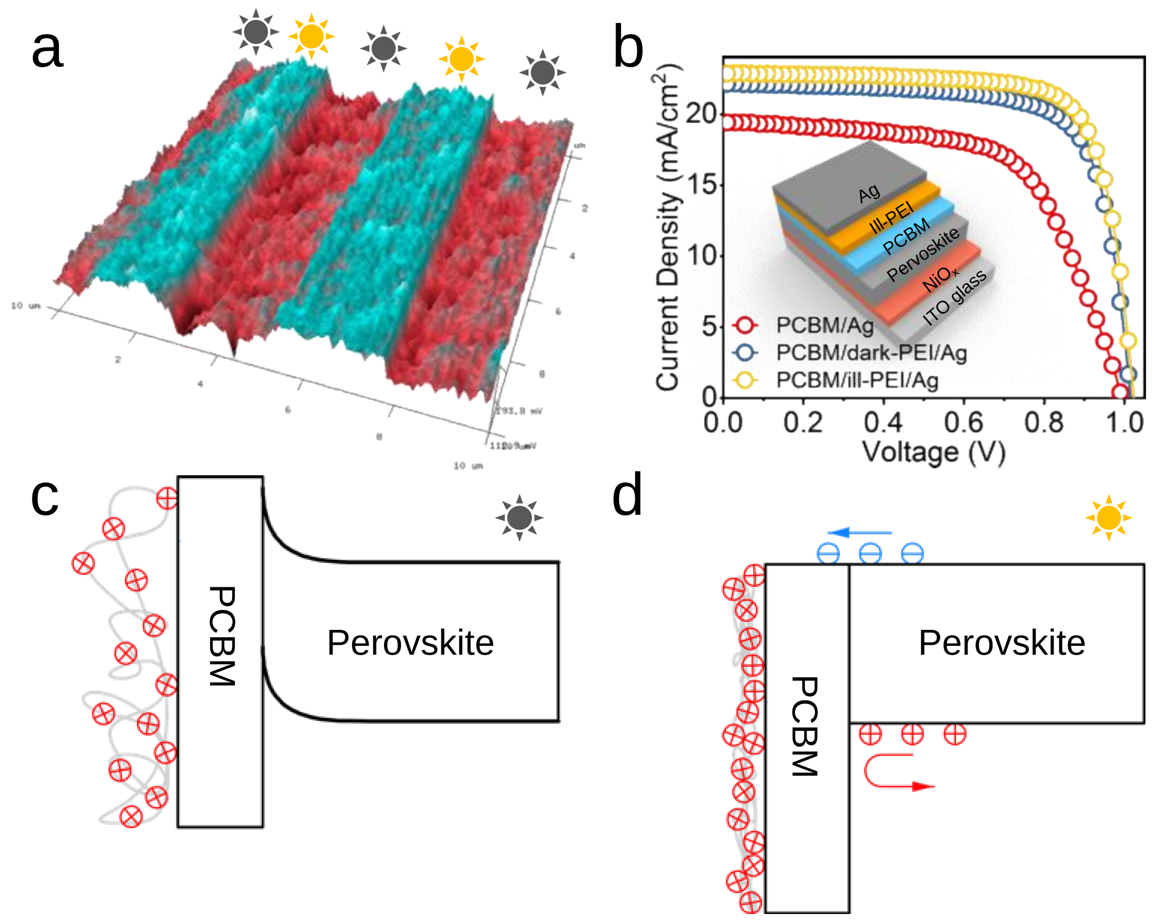

Figure S19. (a) The three-dimensional SKPM image showing instantaneous photoresponse of the perovskite ($MAPbI_3$) when two light ON/OFF cycles were triggered, which resulted in distinct bright and dark bands corresponding to the cycles of light ON and OFF. (b) The light *J–V* curves and photovoltaic parameters of the perovskite solar cells with the dark-PEI and ill-PEI buffer layer between PCBM and Ag electrode. The inset shows the sketch of the perovskite device with the p-i-n structure. The photovoltaic parameters of the cells are listed in the Table S3. Band diagram of the perovskite surface in equilibrium with the PEI solution (c) in dark condition and (d) under illumination.

Supplementary Note 9:

The positive shift of the potential in Figure S18a indicates that the energy level at the surface of the perovskite is upward BB in dark condition as schematically depicted in Figure S18c. Under illumination, a high density of photoexcited electrons and holes are created with in the perovskite film. The BB at the interface of PCBM/PVK is compensated by the V_{SPV},[28] and the photogenerated electrons are easily drawn towards the PCBM (Figure S18d), while the photogenerated holes are blocked by the deep Highest Occupied Molecular Orbital (HOMO) level of PCBM, thus resulting a promoted PEI deposition (here the isopropanol is used as the orthogonal solvents of PEI). It is noted that the structure of the both samples are comparable, therefore we may attribute the observed variation to the difference of the buffer layer between PCBM and Ag. All the PV parameters are improved by the introduction of the ill-PEI buffer layer, which was consistent with our previous results published elsewhere.[29] Both the J_{SC} and the *FF* of the ill-PEI based device are significantly improved due to the reduced film thickness and the increased density of PEI. The V_{OC} enhancement for ill-PEI based device is relatively small, probably because that the V_{OC} is mainly determined by the splitting of quasi-Fermi levels and recombination inside the perovskite rather than the internal electric field established by the difference in the work functions of the two corresponding electrical contacts.[30]

Table S3. Light *J–V* parameters of perovskite solar cells without and with dark-PEI and ill-PEI buffer layer between PCBM and Ag electrode.

**Electron contact**	**PCE** [%]	J_{SC} [mA/cm^2]	V_{OC} [V]	***FF***
PCBM/Ag	11.8 ± 0.4	19.7 ± 0.8	0.98 ± 0.02	0.61± 0.03
PCBM/dark-PEI/Ag	16.6 ± 0.3	22.2 ± 0.8	1.01 ± 0.02	0.74 ± 0.03

PCBM/ill-PEI/Ag	18.0 ± 0.3	22.9 ± 0.7	1.02 ± 0.02	0.77 ± 0.02

[1] J. Gregory, *J. Colloid Interface Sci.* **1981**, *83*, 138.
[2] R. Hogg, T. W. Healy, D. W. Fuerstenau, *Transactions of the Faraday Society* **1966**, *62*, 1638.
[3] P. Bertrand, A. Jonas, A. Laschewsky, R. Legras, *Macromol Rapid Comm* **2000**, *21*, 319.
[4] R. Memming, *Semiconductor Electrochemistry*, Wiley VCH Verlag GmbH, Weinheim **2001**.
[5] K. S. Dieter, *Measurement Science and Technology* **2001**, *12*, R16.
[6] T. Yuan, X. Cui, X. Liu, X. Qu, J. Sun, *Macromolecules* **2019**, *52*, 3141.
[7] H. Kang, S. Hong, J. Lee, K. Lee, *Adv. Mater.* **2012**, *24*, 3005.
[8] R. Schlapak, D. Armitage, N. Saucedo-Zeni, G. Latini, H. J. Gruber, P. Mesquida, Y. Samotskaya, M. Hohage, F. Cacialli, S. Howorka, *Langmuir* **2007**, *23*, 8916.
[9] S. P. Pujari, E. van Andel, O. Yaffe, D. Cahen, T. Weidner, C. J. M. van Rijn, H. Zuilhof, *Langmuir* **2013**, *29*, 570.
[10] D. M. Taylor, O. N. De Oliveira, H. Morgan, *J. Colloid Interface Sci.* **1990**, *139*, 508.
[11] S. Bastide, R. Butruille, D. Cahen, A. Dutta, J. Libman, A. Shanzer, L. Sun, A. Vilan, *J. Phys. Chem. B* **1997**, *101*, 2678.
[12] S. Jeong, S. Jung, H. Kang, D. Lee, S. B. Choi, S. Kim, B. Park, K. Yu, J. Lee, K. Lee, *Adv. Funct. Mater.* **2017**, *27*, 1606842.
[13] R. H. Cox, H. Strack, *Solid State Electron.* **1967**, *10*, 1213.
[14] Y. Wan, C. Samundsett, J. Bullock, T. Allen, M. Hettick, D. Yan, P. Zheng, X. Zhang, J. Cui, J. McKeon, A. Javey, A. Cuevas, *ACS Appl. Mater. Interfaces* **2016**, *8*, 14671.
[15] Y. Wan, C. Samundsett, D. Yan, T. Allen, J. Peng, J. Cui, X. Zhang, J. Bullock, A. Cuevas, *Appl. Phys. Lett.* **2016**, *109*, 113901.
[16] X. Yang, Q. Bi, H. Ali, K. Davis, W. V. Schoenfeld, K. Weber, *Adv. Mater.* **2016**, *28*, 5891.
[17] X. Yang, K. Weber, Z. Hameiri, S. De Wolf, *Prog. Photovolt. Res. Appl.* **2017**, *25*, 896.
[18] A. Richter, J. Benick, F. Feldmann, A. Fell, M. Hermle, S. W. Glunz, *Sol. Energy Mater. Sol. Cells* **2017**, *173*, 96.
[19] J. Cho, J. Melskens, M. Debucquoy, M. R. Payo, S. Jambaldinni, T. Bearda, I. Gordon, J. Szlufcik, W. M. M. Kessels, J. Poortmans, *Prog. Photovolt. Res. Appl.* **2018**, *26*, 835.
[20] Y. Wan, S. K. Karuturi, C. Samundsett, J. Bullock, M. Hettick, D. Yan, J. Peng, P. R. Narangari, S. Mokkapati, H. H. Tan, C. Jagadish, A. Javey, A. Cuevas, *ACS Energy Lett.* **2018**, *3*, 125.
[21] X. Yang, W. Liu, M. De Bastiani, T. Allen, J. Kang, H. Xu, E. Aydin, L. Xu, Q. Bi, H. Dang, E. AlHabshi, K. Kotsovos, A. AlSaggaf, I. Gereige, Y. Wan, J. Peng, C. Samundsett, A. Cuevas, S. De Wolf, *Joule* **2019**, *3*, 1314.
[22] W. Ji, T. Allen, X. Yang, G. Zeng, S. De Wolf, A. Javey, *ACS Energy Lett.* **2020**, *5*, 897.
[23] Y. Wan, C. Samundsett, J. Bullock, M. Hettick, T. Allen, D. Yan, J. Peng, Y. Wu, J. Cui, A. Javey, A. Cuevas, *Adv. Energy Mater.* **2017**, *7*, 1601863.
[24] C. Reichel, U. Würfel, K. Winkler, H.-F. Schleiermacher, M. Kohlstädt, M. Unmüssig, C. A. Messmer, M. Hermle, S. W. Glunz, *J. Appl. Phys.* **2018**, *123*, 024505.
[25] Y. Wan, J. Bullock, M. Hettick, Z. Xu, C. Samundsett, D. Yan, J. Peng, J. Ye, A. Javey, A. Cuevas, *Adv. Energy Mater.* **2018**, *8*, 1800743.
[26] X. Yang, E. Aydin, H. Xu, J. Kang, M. Hedhili, W. Liu, Y. Wan, J. Peng, C. Samundsett, A. Cuevas, S. Wolf, *Adv. Energy Mater.* **2018**, *8*, 1800608.
[27] C. A. Schneider, W. S. Rasband, K. W. Eliceiri, *Nature Methods* **2012**, *9*, 671.
[28] F. Zu, C. M. Wolff, M. Ralaiarisoa, P. Amsalem, D. Neher, N. Koch, *ACS Appl. Mater.*

Interfaces **2019**, *11*, 21578.
[29] Z. Ying, W. Chen, Y. Lin, Z. He, T. Chen, Y. Zhu, X. Zhang, X. Yang, A. B. Djurišić, Z. He, *Adv Opt Mater* **2019**, *7*, 1801409.
[30] S. Ravishankar, S. Gharibzadeh, C. Roldán-Carmona, G. Grancini, Y. Lee, M. Ralaiarisoa, A. M. Asiri, N. Koch, J. Bisquert, M. K. Nazeeruddin, *Joule* **2018**, *2*, 788.
